# Analysis of fractal-fractional Alzheimer's disease mathematical model in sense of Caputo derivative

**DOI:** 10.3934/publichealth.2024020

**Published:** 2024-03-21

**Authors:** Pooja Yadav, Shah Jahan, Kottakkaran Sooppy Nisar

**Affiliations:** 1 Department of Mathematics, Central University of Haryana, Mohindergarh-123031, India; 2 Department of Mathematics, College of Science and Humanities in Alkharj, Prince Sattam Bin Abdulaziz University, Alkharj 11942, Saudi Arabia; 3 Saveetha School of Engineering, SIMATS, Chennai, India

**Keywords:** Alzheimer's disease, fractional Adams-Bashforth method, Ulam-Hyers stability, fractal-fractional derivatives

## Abstract

Alzheimer's disease stands as one of the most widespread neurodegenerative conditions associated with aging, giving rise to dementia and posing significant public health challenges. Mathematical models are considered as valuable tools to gain insights into the mechanisms underlying the onset, progression, and potential therapeutic approaches for AD. In this paper, we introduce a mathematical model for AD that employs the fractal fractional operator in the Caputo sense to characterize the temporal dynamics of key cell populations. This model encompasses essential elements, including amyloid-*β* ($\mathbb{ A_\beta }$), neurons, astroglia and microglia. Using the fractal fractional operator, we have established the existence and uniqueness of solutions for the model under consideration, employing Leray-Schaefer's theorem and the Banach fixed-point methods. Utilizing functional techniques, we have analyzed the proposed model stability under the Ulam-Hyers condition. The suggested model has been numerically simulated by using a fractional Adams-Bashforth approach, which involves a two-step Lagrange polynomial. For numerical simulations, different ranges of fractional order values and fractal dimensions are considered. This new fractal fractional operator in the form of the Caputo derivative was determined to yield better results than an ordinary integer order. Various outcomes are shown graphically by for different fractal dimensions and arbitrary orders.

## 1. Introduction

The World Alzheimer Report (2018) [1] projected that the number of individuals affected by Alzheimer's disease (AD) in 2018 was approximately 50 million, and this figure is anticipated to triple by 2050. AD, a prominent neurodegenerative condition linked to aging, results in dementia, raising significant public health issues [2]. As of now, there remains no known cure for this ailment. Age, gender, and the presence of the e4-allele within the apolipoprotein (APOE4) gene stand as prominent risk factors for AD [3], [4]. Notably, post-menopausal females, especially those carrying the APOE4 gene, exhibit a heightened susceptibility to AD compared to males. Furthermore, AD progression in females appears to manifest at an accelerated pace compared to male counterparts of a similar age. While considerable progress has been made in understanding the roles of age and APOE4 in AD over the past few decades, there remains a notable knowledge gap regarding how gender disparities influence both the onset and course of this debilitating disease.

As females progress from pre-menopause to post-menopause, estradiol levels, the primary estrogen in females, experience a significant decline from 30–400 pg/mL to 0–30 pg/mL. In comparison, normal estradiol levels in males range from 10–50 pg/mL [5]. This research indicates an elevated risk of AD in post-menopausal females compared to males. Growing evidence from genetic, pathological, and functional investigations suggests that an imbalance in the brain's production and clearance of $\mathbb{ A_\beta }$ peptides leads to the accumulation and aggregation of $\mathbb{ A_\beta }$. In addition to the APOE4 gene, other factors influencing the gender bias in AD include hormonal status and glial cell activation (astrocytes and microglia). In their resting state, these cells maintain brain health and in pathological conditions, they transition to an active state. In their resting state, astrocytes regulate synaptic functions and more, while microglia monitor synaptic health. When activated, both cells engage in immune responses to brain pathologies, including AD progression [6], [7].

**Figure 1.**
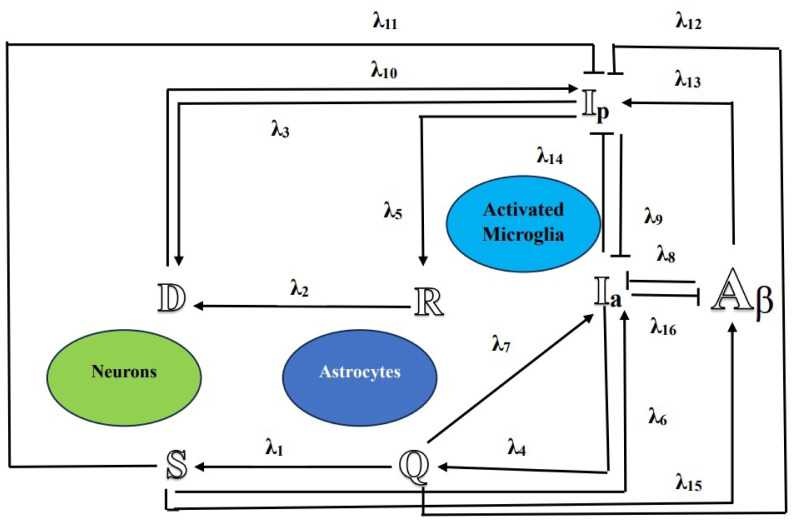
Flowchart of the model.

While the deposition of $\mathbb{ A_\beta }$ peptides and the creation of senile plaques in the brain are primary indicators of AD's clinical phenotype [8], [9], an expanding body of clinical and fundamental research suggests that the inflammatory activation of microglia may hold a comparably significant role in the disease's onset and progression [10]. Microglia, the brain's resident innate immune macrophages, possess the capacity to produce pro-inflammatory substances and reactive oxygen species when triggered by inflammatory signals, including $\mathbb{ A_\beta }$ [11]. In healthy brains, quiescent astroglia ($\mathbb{Q}$), along with resting microglia, can assume an anti-inflammatory state ($\mathbb{I}_a$). This state promotes neuron survival ($\mathbb{S}$) while curbing astroglia proliferation ($\mathbb{R}$) [12]. As inflammatory signals (e.g., $\mathbb{A_\beta}$) accumulate, microglia may shift to an activated pro-inflammatory state ($\mathbb{I}_p$), leading to increased $\mathbb{A_\beta}$ and neuronal death ($\mathbb{D}$) [13]. Moreover, the $\mathbb{I}_a$ phenotype, influenced by estrogen in females [4], undergoes an age-related transition to an $\mathbb{I}_p$-skewed state, which intensifies during the progression of AD [15]. These multiple positive and negative feedback loops among these cell types play a crucial role in the neurodegenerative processes that ultimately impact the structure and function of neurons during AD pathogenesis (see Figure 1).

Mathematical models serve as valuable tools for comprehending the mechanisms underlying AD, encompassing the corresponding onset, progression, and potential therapeutic approaches. While existing mathematical models of AD predominantly address various known features of the disease, including (1) the development of potential AD treatments [16], [17]; (2) the dysrhythmic behavior of inhibitory neurons triggered by AD [18]; (3) the influence of the APOE4 gene on AD onset [19], [20]; (4) the temporal evolution of AD biomarkers [21], [22]; (5) interactions among brain cells and these plaques [23]–[26]; and (6) the formation of $\mathbb{ A_\beta }$ fibrils and plaques [27].

The application of fractional calculus, encompassing both integration and fractional differentiation, offers a more comprehensive insight into real-world challenges than the conventional integer-order calculus. Additionally, it excels at representing and modeling real-world phenomena, primarily owing to its capacity to account for the memory and inherent properties, as substantiated in [28]–[30]. The concept of fractional derivatives lacks a universally accepted definition; instead, it encompasses several distinct formulations, including the Riemann–Liouville, Liouville–Caputo, Grunwald–Letnikov, and other variations. The distinctiveness of fractional derivatives lies in their non-local characteristics, often characterized by exponential decay, power-law behavior, or the presence of Mittag–Leffler kernels. The complexity of mathematical differentiation operators has evolved in conjuction with the increasing complexity of physical problems. Recently, there has been a growing interest in the field of fractal calculus, with several researchers exploring its applications in various scientific and engineering domains [31], [32]. Fractal calculus has introduced a novel approach by combining fractional differentiation with fractal derivatives [38], [39]. It is a powerful technique that allows for a more refined understanding of intricate mathematical models when dealing with real-world data. The fractal fractional derivation is suitable for describing systems with temporal memory and a wide range of spatial influences. Several significant findings have emerged from the application of fractal-fractional operators to solve diverse models in the fields of biology [40], [42] and physics [41], [43]. This emerging field of study has demonstrated its efficacy in tackling complex problems and is poised to make significant contributions to various scientific disciplines [32]–[35].

The objective of this study is to present a mathematical model for AD that extends the framework initially proposed by Puri and Li [23]. We have chosen the model developed by Puri and Li as our foundation due to its ability to capture pathwalks among various cerebral cell populations and the formation of aggregation-prone $\mathbb{ A_\beta }$ fibrils. This model relies on a system of coupled first-order linear ordinary differential equations, with the assumption of a set of constant parameters. In the present work, we have replaced the first order derivative with the fractal-fractional derivative in the Caputo sense. We have examined the model from a different prespective. First, the model is newly constructed, we used the fixed point theory approach to establish existence and uniqueness via the Banach and Leray–Schauder theorems. Second, we employed nonlinear functional analysis to determine conditions for Ulam stability in the system (2.1). We have applied the fractal-fractional operator's basics to achieve intriguing numerical results.

The rest of the article is structured as follows. Section 2, presents the mathematical model of the AD. In Section 3, we establish the existence and uniqueness of the solution through the application of fixed-point theory. Additionally, we also explore the Ulam-Hyers stability within the same section. Moving on to Section 4, we employ the Adams-Bashforth technique to perform numerical simulations and present the graphical results for the analyzed system. Finally, in Section 5, we provide a comprehensive summary of our research findings.

### 1.1. Preliminaries

**Definition 1.1.** *[31] On interval* (a, b)*, let us take $\vartheta(\mathrm{t})$ to be a continuous and differentiable function with* q *order, then the function $\vartheta(\mathrm{t})$ with order* p *of the fractal-fractional derivative in sense of the Riemann-Liouville (R-L) derivative is given by*

1.1 \begin{equation*} 			{ }^{\mathbb{FFP}} \mathfrak{D}^{\mathrm{p,q}} \vartheta(\mathrm{t})=\frac{1}{\Gamma(n-\mathrm{p})} \frac{\mathrm{d}}{\mathrm{dt}^{\mathrm{q}}} \int_0^{\mathrm{t}}(\mathrm{t}-\mathfrak{s})^{n-\mathrm{p}-1} \vartheta(\mathfrak{s}) \mathrm{d}\mathfrak{s}, 		\end{equation*}

$\frac{\mathrm{d} \vartheta(\mathfrak{s})}{\mathrm{d} \mathfrak{s}^{\mathrm{q}}}=\lim _{\mathrm{t} \rightarrow \mathfrak{s}} \frac{\vartheta(\mathrm{t})-\underline{\vartheta}(\mathfrak{s})}{\mathrm{t}^{\mathrm{q}}-\mathfrak{s}^{\mathrm{q}}}$, where $n \in \mathrm{N}$, with $n-1<\mathrm{p}, \mathrm{q} \leqslant n$ .

**Definition 1.2.** *[31] On interval* (a, b)*, take $\vartheta(\mathrm{t})$ as continuous function; then, the function $\vartheta(\mathrm{t})$ with order* p *of the fractal-fractional integral is expressed as:*

1.2 \begin{equation*} 			{ }^{\mathbb{FFP}} \mathrm{I}^{\mathrm{p}} \vartheta(\mathrm{t})=\frac{\mathrm{q}}{\Gamma(\mathrm{p})} \int_0^{\mathrm{t}}\mathfrak{s}^{\mathrm{q}-1}(\mathrm{t}-\mathfrak{s})^{\mathrm{p}-1}  \vartheta(\mathfrak{s}) \mathrm{d}\mathfrak{s} . 		\end{equation*}

## 2. Mathematical model

In [23], the sixteen crosstalks AD mechanism involving seven populations, as shown in Figure 1 are as:

- $\mathbb{A_\beta}$ Aggregation-prone amyloid-*β* fibrils
- $\mathbb{R}$ Proliferative reactive astrocytes
- $\mathbb{Q}$ Quiescent (resting) astrocytes
- $\mathbb{I}_a$ Activated microglia in anti-inflammatory state
- $\mathbb{D}$ Dead neurons
- $\mathbb{S}$ Surviving neurons
- $\mathbb{I}_p$ Activated microglia in pro-inflammatory state

The model operates under the assumption of a consistent risk of neuronal degeneration while overlooking the dispersion of both brain cells and $A_\beta$ fibrils, as previously mentioned. This study presents innovative approache for addressing fractal-fractional problems that have not previously received substantial attention in the literature. The model considered here extends the previous work in [23] in the fractal fractional form:

2.1 \begin{equation*} 		 		\left\{\begin{array}{l}	{ }^{\mathbb{FFP}} \mathfrak{D}^{\mathrm{p,q}} \mathbb{R}(t)=\lambda_5\mathbb{I}_p-\lambda_4\mathbb{I}_a \\ 			 			{ }^{\mathbb{FFP}} \mathfrak{D}^{\mathrm{p,q}} \mathbb{Q}(t)= -\lambda_5\mathbb{I}_p+\lambda_4\mathbb{I}_a\\ 			 			{ }^{\mathbb{FFP}} \mathfrak{D}^{\mathrm{p,q}} \mathbb{A_\beta}(t)= -\lambda_r\mathbb{A_\beta}-\lambda_{16}\mathbb{I}_a+\lambda_{15}\mathbb{S} \\ 			 			{ }^{\mathbb{FFP}} \mathfrak{D}^{\mathrm{p,q}} \mathbb{I}_p(t)= -(\lambda_7+\lambda_{12})\mathbb{Q}+(\lambda_8+\lambda_{13})\mathbb{A_\beta}+\lambda_9\mathbb{I}_p-\lambda_14\mathbb{I}_a-(\lambda_6+\lambda_{11})\mathbb{S}+\lambda_{10}\mathbb{D} \\ 			 			{ }^{\mathbb{FFP}} \mathfrak{D}^{\mathrm{p,q}} \mathbb{I}_a(t)= (\lambda_7+\lambda_{12})\mathbb{Q}-(\lambda_8+\lambda_{13})\mathbb{A_\beta}-\lambda_9\mathbb{I}_p+\lambda_14\mathbb{I}_a+(\lambda_6+\lambda_{11})\mathbb{S}-\lambda_{10}\mathbb{D} \\ 			 			{ }^{\mathbb{FFP}} \mathfrak{D}^{\mathrm{p,q}} \mathbb{S}(t)= -\lambda_2\mathbb{R}+\lambda_1\mathbb{Q}-\lambda_3\mathbb{I}_p \\ 			 			{ }^{\mathbb{FFP}} \mathfrak{D}^{\mathrm{p,q}} \mathbb{D}(t)= =\lambda_2\mathbb{R}-\lambda_1\mathbb{Q}+\lambda_3\mathbb{I}_p\end{array}\right. , 	\end{equation*}

where, the fractal dimension (F-D) q ∈ [0, 1] and the fractional order (F-O) p ∈ [0, 1]. As in [23], the model discussed the parameters values and has provided the sensitivity analysis. The system (2.1) represent multiple signaling pathways detailed in [23]. These pathways which are involve activation (→) and suppression (⊥). For AD inhibition the pathways involved are $\mathbb{Q} \rightarrow \mathbb{I}_a$, $\mathbb{Q} \perp \mathbb{I}_p$, $\mathbb{I}_p \perp \mathbb{I}_a$ and for AD progression the involved pathways are $\mathbb{I}_a \rightarrow \mathbb{D}$, $\mathbb{A_\beta} \perp \mathbb{I}_a$, $\mathbb{A_\beta} \rightarrow \mathbb{I}_p$. Table 1 presents the parameters values of system (2.1).

**Table 1.** Parameters values of system (2.1).

Rate	Pathway	Value (1/year)	Rate	Pathway	Value (1/year)
*λ*_1_	$\mathbb{Q} \rightarrow \mathbb{S}$	10^−5^	*λ*_10_	$\mathbb{D} \rightarrow \mathbb{I}_p$	10^−2^
*λ*_2_	$\mathbb{R} \rightarrow \mathbb{D}$	10^−3^	*λ*_11_	$\mathbb{S} \perp \mathbb{I}_p$	10^−2^
*λ*_3_	$\mathbb{I}_p \rightarrow \mathbb{D}$	10^−2^	*λ*_12_	$\mathbb{Q} \perp \mathbb{I}_p$	10^−4^
*λ*_4_	$\mathbb{I}_a \rightarrow \mathbb{Q}$	10^−4^	*λ*_13_	$\mathbb{A_\beta} \rightarrow \mathbb{I}_a$	10^−2^
*λ*_5_	$\mathbb{I}_p \rightarrow \mathbb{R}$	10^−2^	*λ*_14_	$\mathbb{I}_a \perp \mathbb{I}_p$	10^−4^
*λ*_6_	$\mathbb{S} \rightarrow \mathbb{I}_a$	10^−2^	*λ*_15_	$\mathbb{S} \rightarrow \mathbb{A_\beta}$	1
*λ*_7_	$\mathbb{Q} \rightarrow \mathbb{I}_a$	10^−4^	*λ*_16_	$\mathbb{I}_a \perp \mathbb{A_\beta}$	10^−2^
*λ*_8_	$\mathbb{A_\beta} \perp \mathbb{I}_a$	10^−2^	*λ_r_*	$\mathbb{I}_a \perp \mathbb{A_\beta}$	1
*λ*_9_	$\mathbb{I}_p \perp \mathbb{I}_a$	10^−2^			

## 3. Existence and uniqueness analysis of the model

Before we start investigating the biological model, it is important to check if such a dynamic problem really exists in the real world. To check the existence and uniqueness of system (2.1), we can use a theory called the fixed point theory. In this study, we chose to apply this theory to our proposed model (2.1). Consider the Banach space $\mathscr{V}=\mathscr{W} \times \mathscr{W} \times \mathscr{W} \times$ $\mathscr{W} \times \mathscr{W} \times \mathscr{W} \times \mathscr{W}$, where $\mathscr{W}=C(\mathscr{I})$; $\|\mathscr{Z}\|=\|\mathbb{R}, \mathbb{Q}, \mathbb{A_\beta}, \mathbb{I}_p, \mathbb{I}_a, \mathbb{S}, \mathbb{D}\|=\quad \max _{\mathrm{t} \in \mathscr{W}}[|\mathbb{R}(\mathrm{t})|+$ $|\mathbb{Q}(\mathrm{t})|+|\mathbb{A_\beta}(\mathrm{t})|+|\mathbb{I}_p(\mathrm{t})|+|\mathbb{I}_a(\mathrm{t})|+|\mathbb{S}(\mathrm{t})|+|\mathbb{D}(\mathrm{t})|]$.

As the integral can be differentiated, we can express the initial problem (model (2.1)) as follows:

3.1 \begin{equation*} 		\left\{\begin{array}{l}	{ }^{\mathbb{RL}} \mathfrak{D}^{\mathrm{p}} \mathbb{R}(t)=qt^{q-1}h_1(t, \mathbb{R}, \mathbb{Q}, \mathbb{A_\beta}, \mathbb{I}_p, \mathbb{I}_a, \mathbb{S}, \mathbb{D}) \\ 			{ }^{\mathbb{RL}} \mathfrak{D}^{\mathrm{p}} \mathbb{Q}(t)=qt^{q-1}h_2(t, \mathbb{R}, \mathbb{Q}, \mathbb{A_\beta}, \mathbb{I}_p, \mathbb{I}_a, \mathbb{S}, \mathbb{D}) \\ 			{ }^{\mathbb{RL}} \mathfrak{D}^{\mathrm{p}} \mathbb{A_\beta}(t)=qt^{q-1}h_3(t, \mathbb{R}, \mathbb{Q}, \mathbb{A_\beta}, \mathbb{I}_p, \mathbb{I}_a, \mathbb{S}, \mathbb{D}) \\			 			{ }^{\mathbb{RL}} \mathfrak{D}^{\mathrm{p}} \mathbb{I}_p(t)=qt^{q-1}h_4(t, \mathbb{R}, \mathbb{Q}, \mathbb{A_\beta}, \mathbb{I}_p, \mathbb{I}_a, \mathbb{S}, \mathbb{D}) \\ 			{ }^{\mathbb{RL}} \mathfrak{D}^{\mathrm{p}} \mathbb{I}_a(t)=qt^{q-1}h_5(t, \mathbb{R}, \mathbb{Q}, \mathbb{A_\beta}, \mathbb{I}_p, \mathbb{I}_a, \mathbb{S}, \mathbb{D}) \\ 			{ }^{\mathbb{RL}} \mathfrak{D}^{\mathrm{p}} \mathbb{S}(t)=qt^{q-1}h_6(t, \mathbb{R}, \mathbb{Q}, \mathbb{A_\beta}, \mathbb{I}_p, \mathbb{I}_a, \mathbb{S}, \mathbb{D}) \\ 			{ }^{\mathbb{RL}} \mathfrak{D}^{\mathrm{p}} \mathbb{D}(t)=qt^{q-1}h_7(t, \mathbb{R}, \mathbb{Q}, \mathbb{A_\beta}, \mathbb{I}_p, \mathbb{I}_a, \mathbb{S}, \mathbb{D})\end{array}\right. , 	\end{equation*}

where

3.2 \begin{equation*} 		\left\{\begin{array}{l}h_1(t, \mathbb{R}, \mathbb{Q}, \mathbb{A_\beta}, \mathbb{I}_p, \mathbb{I}_a, \mathbb{S}, \mathbb{D})=\lambda_5\mathbb{I}_p-\lambda_4\mathbb{I}_a \\  			h_2(t, \mathbb{R}, \mathbb{Q}, \mathbb{A_\beta}, \mathbb{I}_p, \mathbb{I}_a, \mathbb{S}, \mathbb{D})=-\lambda_5\mathbb{I}_p+\lambda_4\mathbb{I}_a \\ h_3(t, \mathbb{R}, \mathbb{Q}, \mathbb{A_\beta}, \mathbb{I}_p, \mathbb{I}_a, \mathbb{S}, \mathbb{D})=-\lambda_r\mathbb{A_\beta}-\lambda_{16}\mathbb{I}_a+\lambda_{15}\mathbb{S} \\ h_4(t, \mathbb{R}, \mathbb{Q}, \mathbb{A_\beta}, \mathbb{I}_p, \mathbb{I}_a, \mathbb{S}, \mathbb{D})=-(\lambda_7+\lambda_{12})\mathbb{Q}+(\lambda_8+\lambda_{13})\mathbb{A_\beta}+\lambda_9\mathbb{I}_p-\lambda_14\mathbb{I}_a-(\lambda_6+\lambda_{11})\mathbb{S}+\lambda_{10}\mathbb{D} \\ h_5(t, \mathbb{R}, \mathbb{Q}, \mathbb{A_\beta}, \mathbb{I}_p, \mathbb{I}_a, \mathbb{S}, \mathbb{D})=(\lambda_7+\lambda_{12})\mathbb{Q}-(\lambda_8+\lambda_{13})\mathbb{A_\beta}-\lambda_9\mathbb{I}_p+\lambda_14\mathbb{I}_a+(\lambda_6+\lambda_{11})\mathbb{S}-\lambda_{10}\mathbb{D} \\ h_6(t, \mathbb{R}, \mathbb{Q}, \mathbb{A_\beta}, \mathbb{I}_p, \mathbb{I}_a, \mathbb{S}, \mathbb{D})=-\lambda_2\mathbb{R}+\lambda_1\mathbb{Q}-\lambda_3\mathbb{I}_p \\ h_7(t, \mathbb{R}, \mathbb{Q}, \mathbb{A_\beta}, \mathbb{I}_p, \mathbb{I}_a, \mathbb{S}, \mathbb{D}) =\lambda_2\mathbb{R}-\lambda_1\mathbb{Q}+\lambda_3\mathbb{I}_p\end{array}\right. . 	\end{equation*}

Utilizing equation (3.1) and considering the domain of t within the set $\mathscr{I}$, we can represent the developed system as follows:

3.3 \begin{equation*} 		\begin{aligned} 			{ }^{\mathbb{RL}} \mathfrak{D}^{\mathrm{p}} \mathscr{Y}(t) & =\mathrm{qt}^{\mathrm{q}-1} \Theta(t, \mathscr{Y}(t)), \quad 0<\mathrm{p}, \mathrm{q} \leq 1, \\ 			\mathscr{Y}(0) & =\mathscr{Y}_0 . 		\end{aligned} 	\end{equation*}

By substituting ${ }^{\mathbb{RL}} \mathfrak{D}^{\mathrm{p}, \mathrm{q}}$ with ${ }^{\mathbb{C}} \mathfrak{D}^{\mathrm{p}, \mathrm{q}}$ and employing the R-L type integral, we can derive the solution for equation (3.3) as follows:

3.4 \begin{equation*} 		\mathscr{Y}(t)=\mathscr{Y}_0(t)+\frac{\mathrm{q}}{\Gamma(\mathrm{p})} \int_0^{t} \mathfrak{s}^{\mathrm{q}-1}(\mathrm{t}-\mathfrak{s})^{\mathrm{p}-1} \Theta(\mathfrak{s}, \mathscr{Y}(\mathfrak{s})) \mathrm{d}\mathfrak{s}, 	\end{equation*}

where $\mathscr{Y}(t)=\left\{\begin{array}{l} \mathbb{R}(t)\\ \mathbb{Q}(t)\\ \mathbb{A_\beta}(t)\\ \mathbb{I}_p(t)\\ \mathbb{I}_a(t)\\ \mathbb{S}(t)\\ \mathbb{D}(t)\end{array} \quad \mathscr{Y}_0(\mathrm{t})=\left\{\begin{array}{l}\mathbb{R}_{0}\\ \mathbb{Q}_{0}\\ \mathbb{A_\beta}_{0}\\ \mathbb{I}_{p_{0}}\\ \mathbb{I}_{a_{0}}\\ \mathbb{S}_{0}\\ \mathbb{D}_{0}\end{array}, \quad \mathbb{\Theta}(\mathrm{t}, \mathscr{Y}(\mathrm{t}))=\left\{\begin{array}{l}h_1(t, \mathbb{R}, \mathbb{Q}, \mathbb{A_\beta}, \mathbb{I}_p, \mathbb{I}_a, \mathbb{S}, \mathbb{D}) \\ h_2(t, \mathbb{R}, \mathbb{Q}, \mathbb{A_\beta}, \mathbb{I}_p, \mathbb{I}_a, \mathbb{S}, \mathbb{D})\\ h_3(t, \mathbb{R}, \mathbb{Q}, \mathbb{A_\beta}, \mathbb{I}_p, \mathbb{I}_a, \mathbb{S}, \mathbb{D})\\ h_4(t, \mathbb{R}, \mathbb{Q}, \mathbb{A_\beta}, \mathbb{I}_p, \mathbb{I}_a, \mathbb{S}, \mathbb{D}) \\ h_5(t, \mathbb{R}, \mathbb{Q}, \mathbb{A_\beta}, \mathbb{I}_p, \mathbb{I}_a, \mathbb{S}, \mathbb{D}) \\h_6(t, \mathbb{R}, \mathbb{Q}, \mathbb{A_\beta}, \mathbb{I}_p, \mathbb{I}_a, \mathbb{S}, \mathbb{D}) \\ h_7(t, \mathbb{R}, \mathbb{Q}, \mathbb{A_\beta}, \mathbb{I}_p, \mathbb{I}_a, \mathbb{S}, \mathbb{D})\end{array}\right.\right.\right.$

Now, transform the problem (2.1) into the fixed point problem. Consider the operator $\mathbf{Z}: \mathscr{V} \rightarrow \mathscr{V}$ defined by:

3.5 \begin{equation*} 		\mathbf{Z}(\mathscr{Y})(\mathrm{t})=\mathscr{Y}_0(\mathrm{t})+\frac{\mathrm{q}}{\Gamma(\mathrm{p})} \int_0^{\mathrm{t}} \mathfrak{s}^{\mathrm{q}-1}(\mathrm{t}-\mathfrak{s})^{\mathrm{p}-1} \Theta(\mathfrak{s}, \mathscr{Y}(\mathfrak{s})) \mathrm{d}\mathfrak{s}. 	\end{equation*}

For the existence of the considered model, the following theorem is used [14].

**Theorem 3.1.** *Let us take a completely continuouse operator $\mathbf{Z}: \mathscr{V} \rightarrow \mathscr{V}$ and consider the set defined by:*

$$\mathscr{A}(\mathbf{Z})=\{\mathscr{Y} \in \mathscr{V}: \mathscr{V}=\theta \mathbf{Z}(\mathscr{Y}), \theta \in[0,1]\}$$

be bounded. Then **Z** has a fixed point in $\mathscr{V}$.

**Theorem 3.2.** *Consider a continuous function $\Theta: \mathscr{I} \times \mathscr{V} \rightarrow \mathrm{R}$. Then the* **Z** *operator is compact*.

*Proof*. Take a bounded set **A** in $\mathscr{V}$. So, there exists $\mathscr{C}_{\Theta}>0$ with ?$|\Theta(\mathrm{t}, \mathscr{Y}(\mathrm{t}))| \leqslant \mathscr{C}_{\Theta}, \forall \mathscr{Y} \in \mathbf{A}$. For any $\mathscr{Y} \in \mathbf{A}$, we get

3.6 \begin{equation*} 			\begin{aligned} 				\|\mathbf{Z}(\mathscr{Y})\| &\leqslant \frac{\mathrm{ q} \mathscr{C}_{\Theta}}{\Gamma(\mathrm{p})} \max _{1 \rightarrow \mathcal{I}} \int_0^{\mathrm{t}} \mathfrak{s}^{\mathrm{q}-1}(\mathrm{t}-\mathfrak{s})^{\mathrm{q}-1} \mathrm{d}\mathfrak{s}, \\ 				&  \frac{\mathrm{ q} \mathscr{C}_{\Theta}}{\Gamma(\mathrm{p})} \max _{1 \rightarrow \mathcal{I}} \int_0^1 \mathfrak{s}^{\mathrm{p}-1}(1-\mathfrak{s})^{\mathrm{q}-1} t^{\mathrm{p}+\mathrm{q}-1} d \mathfrak{s} \text {, } \\ 				& \leqslant \frac{\mathrm{q} \mathscr{C}_{\Theta}\mathbf{Z}^{\mathrm{p}+\mathrm{q}-1}}{\Gamma(\mathrm{p})} \mathbb{B}(\mathrm{p,q}), \\ 				& 			\end{aligned} 		\end{equation*}

where $\mathbb{B}(\mathrm{p,q})$ is the beta function. Thus, (3.6) implies that **Z** is uniformly bounded.

Subsequently, to establish the equicontinuity property of the operator **Z**, considering any two points t_1_ and t_2_ within the interval $\mathscr{I}$, where $\mathscr{I}$ belongs to the set **A**, we observe the following:

$$ 		\begin{aligned} 			\|\mathbf{Z}(\mathscr{Y})(t_1)-\mathbf{Z}(\mathscr{Y})(t_2)\|	& \leqslant \dfrac{q\mathscr{C}}{\Gamma(p)}\max _{t \rightarrow \mathscr{I}}|\int_{0}^{t_1}(t_1-\mathfrak{s})^{p-1}\mathfrak{s}^{q-1}d\mathfrak{s}-\int_{0}^{t_2}(t_2-\mathfrak{s})^{p-1}\mathfrak{s}^{q-1}d\mathfrak{s}|,\\ 			&\leqslant \dfrac{q\mathscr{C}_\Theta \mathbb{B}(\mathrm{p,q})}{\Gamma(p)}(t_{1}^{p-1+q}-t_{2}^{p-1+q})\rightarrow 0 \quad  \quad (t_1 \rightarrow t_2). 		\end{aligned} 		$$

So, **Z** is equicontinuous. Therefore, **Z** is both a continuous operator and bounded, hence, by the Arzelá-Ascoli theorem **Z** is completely continuous and relatively compact.    □

**Theorem 3.3.** *Suppose that $\forall$ $\mathrm{t} \in \mathscr{I}$ and $\mathscr{Y} \in \mathrm{R}$, there exists a positive real number $\mathscr{C}_{\Theta}>0$ such that $|\Theta(\mathrm{t}, \mathscr{Y}(\mathrm{t}))| \leqslant \mathscr{C}_{\Theta}$. Under these conditions, the model (2.1) has at least one solution within the specified space $\mathscr{V}$*.

*Proof*. Let, a set $\mathscr{A}=\{\mathscr{Y} \in \mathscr{V}: \mathscr{Y}=\theta \mathbf{Z}(\mathscr{Y}), \theta \in[0,1]\}$ and show that $\mathscr{A}$ is bounded. Consider $\mathscr{Y} \in \mathscr{A}$, then, $\mathscr{Y}=\theta \mathbf{Z}(\mathscr{Y})$. For $\mathrm{t} \in \mathscr{I}$, we get

$$ 		\|\mathscr{Y}\| \leqslant \frac{\mathrm{q} \mathscr{C}_\Theta \mathrm{Z}^{\mathrm{p}+\mathrm{q}-1}}{\Gamma(\mathrm{p})} \mathbb{B}(\mathrm{p}, \mathrm{q}) . 		$$

Therefore, $\mathscr{A}$ is bounded. **Z** has at least one fixed point given by Theorem 3.1,3.2. Hence, model (2.1) has at least one solution.

For a more in-depth analysis, let us consider the following hypothesis:

($\mathbb{H}$) There exists a constant $\mathscr{X}_{\Theta}>0$ such that for any $\mathscr{Y}, \overline{\mathscr{Y}} \in \mathscr{V}$, the following inequality holds:

$$ 		|\Theta(\mathrm{t}, \mathscr{Y})-\Theta(\mathrm{t}, \overline{\mathscr{Y}})| \leqslant \mathscr{X}_{\Theta}|\mathscr{Y}-\overline{\mathscr{Y}}| . 		$$

To establish uniqueness, we will employ Banach's contraction Theorem [14].   □

**Theorem 3.4.** *Assuming that condition ($\mathbb{H}$) holds true and if $\Xi$ < 1, then the solution to the given model (2.1) is unique*.

3.7 \begin{equation*} 			\Xi=\frac{\mathrm{ q} \mathscr{X}_\Theta \mathbf{Z}^{\mathrm{ p}+\mathrm{ q}-1}}{\Gamma(\mathrm{ q})} \mathbb{B}(\mathrm{ p}, \mathrm{ q}) . 		\end{equation*}

*Proof*. Define $\max _{t \in \mathscr{I}}|\Theta(\mathrm{t}, 0)|=\mathscr{G}_{\Theta}<\infty$, such that

$$ 		\mathrm{h} \geqslant \frac{\mathrm{q} \mathbf{Z}^{\mathrm{p}+\mathrm{q}-1} \mathbb{B}(\mathrm{p}, \mathrm{q}) \mathscr{G}_\Theta}{\Gamma(\mathrm{p})-\mathrm{q} \mathbf{Z}^{\mathrm{p}+\mathrm{q}-1} \mathbb{B}(\mathrm{p}, \mathrm{q}) \mathscr{X}_\Theta} 		$$

We prove that $\mathbf{Z}\left(\mathfrak{A}_h\right) \subset \mathfrak{A}_{\mathrm{h}}$, where $\mathfrak{A}_{\mathrm{h}}=\{\mathscr{Y} \in \mathscr{V}:\|\mathscr{Y}\| \leqslant \mathrm{h}\}$. For $\mathscr{Y} \in \mathfrak{A}_\mathrm{h}$, we obtain

$$ 		\begin{aligned} 			\|\mathbf{Z}(\mathscr{Y})\|	& \leqslant \dfrac{\mathrm{q}}{\Gamma(\mathrm{p})}\max _{t \rightarrow \mathscr{I}}\int_{0}^{t}(t-\mathfrak{s})^{\mathrm{p}-1}\mathfrak{s}^{\mathrm{q}-1}(|\Theta(t,0)|+|\Theta(t,\mathscr{Y}(t))-\Theta(t,0)|)d\mathfrak{s},\\ 			&\leqslant \frac{\mathrm{q}\mathbf{Z}^{\mathrm{p}+\mathrm{q}-1}\mathbb{B}(\mathrm{p,q})(\mathscr{Y}_\Theta)\|\mathscr{Y}\|+\mathscr{G}_\Theta}{\Gamma(\mathrm{p})}.\\ 			&\leqslant \frac{\mathrm{q}\mathbf{Z}^{\mathrm{p}+\mathrm{q}-1}\mathbb{B}(\mathrm{p,q})(\mathscr{Y}_\Theta)r+\mathscr{G}_\Theta}{\Gamma(\mathrm{p})}.\\ 			&\mathrm{h}. 		\end{aligned} 		$$

Consider the operator $\mathbf{Z}: \mathscr{V} \rightarrow \mathscr{V}$ defined by (3.5). Using ($\mathbb{H}$), for $\mathscr{Y}, \overline{\mathscr{Y}} \in \mathscr{\mathscr { V }}$ and for all $\mathrm{t} \in \mathscr{I}$, we therefore have

3.8 \begin{equation*} 			\begin{aligned} 				\|\mathbf{Z}(\mathscr{Y})-\mathbf{Z}(\mathscr{\bar{Y}})\|	& \leqslant \dfrac{q}{\Gamma(p)}\max _{t \rightarrow \mathscr{I}}|\int_{0}^{t}(t-\mathfrak{s})^{p-1}\mathfrak{s}^{q-1}\Theta(\mathfrak{s},\mathscr{Y}(\mathfrak{s}))d\mathfrak{s}-\int_{0}^{t}(t-\mathfrak{s})^{p-1}\mathfrak{s}^{q-1}\Theta(\mathfrak{s},\bar{\mathscr{Y}}(\mathfrak{s}))d\mathfrak{s}|,\\ 				&\leqslant \Xi\|\mathscr{Y}-\mathscr{\bar{Y}}\| 			\end{aligned} 		\end{equation*}

Therefore, we can conclude that **Z** satisfies the contraction condition as in (3.8). Consequently, the integral equation (3.4) possesses a unique solution. Hence, we conclude that it holds true for model (2.1).   □

## 4. Ulam stability

Here, we will study the model (2.1) stability by taking $\Phi \in C(\mathscr{I})$ (small perturbation). This change depends only on Φ(0) = 0 and the solution. Next, considering the following:

- for $\epsilon>0$, $|\Phi(\mathrm{t})| \leqslant \epsilon$
- $ { }^{\mathbb{FFP}} \mathfrak{D}^{\mathrm{p,q}} \mathscr{Y}(\mathrm{t})=\Phi(\mathrm{t})+\Theta(\mathrm{t}, \mathscr{Y}(\mathrm{t}))$.

**Lemma 4.1.** *The perturbed problem solution will be*

$$ 		\begin{aligned} 			{ }^{\mathbb{FFP}} \mathfrak{D}^{\mathrm{p,q}}\mathscr{Y}(\mathrm{t}) & =\Theta(\mathrm{t}, \mathscr{Y}(\mathrm{t}))+\Phi(\mathrm{t}) \\ 			\mathscr{Y}(0) & =\mathscr{Y}_0 		\end{aligned} 		$$

satisfying

4.1 \begin{equation*} 			|\mathscr{Y}(t)-(\frac{q}{\Gamma(p)}\int_{0}^{t}(t-\mathfrak{s})^{p-1}\mathfrak{s}^{q-1}\Theta(\mathfrak{s},\mathscr{Y}(\mathfrak{s}))d\mathfrak{s}+\mathscr{Y}_0(t))|\leqslant \left(\frac{\mathrm{q}\mathbf{Z}^{\mathrm{p}+\mathrm{q}-1}}{\Gamma(\mathrm{p})}\mathbb{B}(p,q)\right)\epsilon=\mathbb{C}_{\mathrm{p, q}}\epsilon. 		\end{equation*}

**Theorem 4.2.** *Considering the assumptions $\mathbb{H}$ and (4.1) in Lemma 4.1, we find that the solution to integral equation (3.4) exhibits Ulam-Hyers stability. As a result, we can conclude that the entire system under consideration attains Ulam-Hyers stability when $\Xi$ (as defined in (3.7)) <* 1.

*Proof*. Consider a unique solution $\mathscr{L} \in \mathscr{V}$ and $\mathscr{Y} \in \mathscr{V}$ to be any solution of (3.4), so

$$ 		\begin{aligned} 			|\mathscr{Y}(t)-\mathscr{L}(t)|&=|\mathscr{Y}(t)-(\mathscr{L}_0(t)+\frac{q}{\Gamma(p)}\int_{0}^{t}(t-\mathfrak{s})^{p-1}\mathfrak{s}^{q-1}\Theta(\mathfrak{s},\mathscr{L}(\mathfrak{s}))d\mathfrak{s})|  \\ 			&\leqslant |\mathscr{Y}(t)-(\mathscr{Y}_0(t)+\frac{q}{\Gamma(p)}\int_{0}^{t}(t-\mathfrak{s})^{p-1}\mathfrak{s}^{q-1}\Theta(\mathfrak{s},\mathscr{Y}(\mathfrak{s}))d\mathfrak{s})|\\ 			&+|(\mathscr{Y}_0(t)+\frac{q}{\Gamma(p)}\int_{0}^{t}(t-\mathfrak{s})^{p-1}\mathfrak{s}^{q-1}\Theta(\mathfrak{s},\mathscr{Y}(\mathfrak{s}))d\mathfrak{s})\\ 			&-(\mathscr{L}_0(t)+\frac{q}{\Gamma(p)}\int_{0}^{t}(t-\mathfrak{s})^{p-1}\mathfrak{s}^{q-1}\Theta(\mathfrak{s},\mathscr{L}(\mathfrak{s}))d\mathfrak{s})|\\ 			&\leqslant \mathbb{C}_{p,q}\epsilon+\frac{q\mathscr{X}\mathbf{Z}^{p+q-1}\mathbb{B}(p,q)}{\Gamma(p)}\|\mathscr{Y}-\mathscr{L}\|. 		\end{aligned} 		$$

From this we obtain

4.2 \begin{equation*} 			\|\mathscr{Y}-\mathscr{L}\| \leqslant \mathrm{C}_{p,q}\epsilon+\Xi\|\mathscr{Y}-\mathscr{L}\|. 		\end{equation*}

From (4.2), we get

4.3 \begin{equation*} 			\|\mathscr{Y}-\mathscr{L}\| \leqslant \left(\frac{\mathbb{C}_{p,q}}{1-\Xi}\right) \epsilon. 		\end{equation*}

Therefore, the outcome derived from equation (4.3) implies that the solution to equation (3.4) exhibits Ulam-Hyers stability. As a result, we can conclude that the solution to the given problem also demonstrates Ulam-Hyers stability.   □

## 5. Numerical scheme

A numerical algorithm is presented here for the model (2.1) for subsequent implementation in numerical simulations. Specifically, for our numerical approach, we will outline the formulation of (3.4) of the model under consideration in the following manner:

5.1 \begin{equation*} 		\left\{\begin{array}{l} 			\mathbb{R}=\mathbb{R}_0+\frac{\mathrm{q}}{\Gamma(\mathrm{p})} \int_0^{\mathrm{t}} \mathfrak{s}^{\mathrm{q}-1}\left(\mathrm{t}-\mathfrak{s}\right)^{\mathrm{p}-1} h_1(\mathfrak{s}, \mathbb{R}(\mathfrak{s}), \mathbb{Q}(\mathfrak{s}), \mathbb{A_\beta}(\mathfrak{s}), \mathbb{I}_p(\mathfrak{s}), \mathbb{I}_a(\mathfrak{s}), \mathbb{S}(\mathfrak{s}), \mathbb{D}(\mathfrak{s})) \mathrm{d} \mathfrak{s}, \\ 			\mathbb{Q}=\mathbb{Q}_0+\frac{\mathrm{q}}{\Gamma(\mathrm{p})} \int_0^{\mathrm{t}} \mathfrak{s}^{\mathrm{q}-1}\left(\mathrm{t}-\mathfrak{s}\right)^{\mathrm{p}-1} h_2(\mathfrak{s}, \mathbb{R}(\mathfrak{s}), \mathbb{Q}(\mathfrak{s}), \mathbb{A_\beta}(\mathfrak{s}), \mathbb{I}_p(\mathfrak{s}), \mathbb{I}_a(\mathfrak{s}), \mathbb{S}(\mathfrak{s}), \mathbb{D}(\mathfrak{s})) \mathrm{d} \mathfrak{s},  \\ 			\mathbb{A_\beta}=\mathbb{A_\beta}_0+\frac{\mathrm{q}}{\Gamma(\mathrm{p})} \int_0^{\mathrm{t}} \mathfrak{s}^{\mathrm{q}-1}\left(\mathrm{t}-\mathfrak{s}\right)^{\mathrm{p}-1} h_3(\mathfrak{s}, \mathbb{R}(\mathfrak{s}), \mathbb{Q}(\mathfrak{s}), \mathbb{A_\beta}(\mathfrak{s}), \mathbb{I}_p(\mathfrak{s}), \mathbb{I}_a(\mathfrak{s}), \mathbb{S}(\mathfrak{s}), \mathbb{D}(\mathfrak{s})) \mathrm{d} \mathfrak{s},  \\ 			\mathbb{I}_p=\mathbb{I}_{p_0}+\frac{\mathrm{q}}{\Gamma(\mathrm{p})} \int_0^{\mathrm{t}} \mathfrak{s}^{\mathrm{q}-1}\left(\mathrm{t}-\mathfrak{s}\right)^{\mathrm{p}-1} h_4(\mathfrak{s}, \mathbb{R}(\mathfrak{s}), \mathbb{Q}(\mathfrak{s}), \mathbb{A_\beta}(\mathfrak{s}), \mathbb{I}_p(\mathfrak{s}), \mathbb{I}_a(\mathfrak{s}), \mathbb{S}(\mathfrak{s}), \mathbb{D}(\mathfrak{s})) \mathrm{d} \mathfrak{s}, \\ 			\mathbb{I}_a=\mathbb{I}_{a_0}+\frac{\mathrm{q}}{\Gamma(\mathrm{p})} \int_0^{\mathrm{t}} \mathfrak{s}^{\mathrm{q}-1}\left(\mathrm{t}-\mathfrak{s}\right)^{\mathrm{p}-1} h_5(\mathfrak{s}, \mathbb{R}(\mathfrak{s}), \mathbb{Q}(\mathfrak{s}), \mathbb{A_\beta}(\mathfrak{s}), \mathbb{I}_p(\mathfrak{s}), \mathbb{I}_a(\mathfrak{s}), \mathbb{S}(\mathfrak{s}), \mathbb{D}(\mathfrak{s})) \mathrm{d} \mathfrak{s},  \\ 			\mathbb{S}=\mathbb{S}_0+\frac{\mathrm{q}}{\Gamma(\mathrm{p})} \int_0^{\mathrm{t}} \mathfrak{s}^{\mathrm{q}-1}\left(\mathrm{t}-\mathfrak{s}\right)^{\mathrm{p}-1} h_6(\mathfrak{s}, \mathbb{R}(\mathfrak{s}), \mathbb{Q}(\mathfrak{s}), \mathbb{A_\beta}(\mathfrak{s}), \mathbb{I}_p(\mathfrak{s}), \mathbb{I}_a(\mathfrak{s}), \mathbb{S}(\mathfrak{s}), \mathbb{D}(\mathfrak{s})) \mathrm{d} \mathfrak{s},  \\ 			\mathbb{D}=\mathbb{D}_0+\frac{\mathrm{q}}{\Gamma(\mathrm{p})} \int_0^{\mathrm{t}} \mathfrak{s}^{\mathrm{q}-1}\left(\mathrm{t}-\mathfrak{s}\right)^{\mathrm{p}-1} h_7(\mathfrak{s}, \mathbb{R}(\mathfrak{s}), \mathbb{Q}(\mathfrak{s}), \mathbb{A_\beta}(\mathfrak{s}), \mathbb{I}_p(\mathfrak{s}), \mathbb{I}_a(\mathfrak{s}), \mathbb{S}(\mathfrak{s}), \mathbb{D}(\mathfrak{s})) \mathrm{d} \mathfrak{s}, . 		\end{array}\right. 	\end{equation*}

By using a new approach at *t_m_*_+1_, we present the numerical solution to (5.1). So, we obtain

$$ 	\left\{\begin{array}{l} 		\mathbb{R}_{m+1}=\mathbb{R}_0+\frac{\mathrm{q}}{\Gamma(\mathrm{p})} \int_0^{\mathrm{t_{m+1}}} \mathfrak{s}^{\mathrm{q}-1}\left(\mathrm{t_{m+1}}-\mathfrak{s}\right)^{\mathrm{p}-1} h_1(\mathfrak{s}, \mathbb{R}(\mathfrak{s}), \mathbb{Q}(\mathfrak{s}), \mathbb{A_\beta}(\mathfrak{s}), \mathbb{I}_p(\mathfrak{s}), \mathbb{I}_a(\mathfrak{s}), \mathbb{S}(\mathfrak{s}), \mathbb{D}(\mathfrak{s})) \mathrm{d} \mathfrak{s},  \\ 		\mathbb{Q}_{m+1}=\mathbb{Q}_0+\frac{\mathrm{q}}{\Gamma(\mathrm{p})} \int_0^{\mathrm{t_{m+1}}} \mathfrak{s}^{\mathrm{q}-1}\left(\mathrm{t_{m+1}}-\mathfrak{s}\right)^{\mathrm{p}-1} h_2(\mathfrak{s}, \mathbb{R}(\mathfrak{s}), \mathbb{Q}(\mathfrak{s}), \mathbb{A_\beta}(\mathfrak{s}), \mathbb{I}_p(\mathfrak{s}), \mathbb{I}_a(\mathfrak{s}), \mathbb{S}(\mathfrak{s}), \mathbb{D}(\mathfrak{s})) \mathrm{d} \mathfrak{s},  \\ 		\mathbb{A_\beta}_{m+1}=\mathbb{A_\beta}_0+\frac{\mathrm{q}}{\Gamma(\mathrm{p})} \int_0^{\mathrm{t_{m+1}}} \mathfrak{s}^{\mathrm{q}-1}\left(\mathrm{t_{m+1}}-\mathfrak{s}\right)^{\mathrm{p}-1} h_3(\mathfrak{s}, \mathbb{R}(\mathfrak{s}), \mathbb{Q}(\mathfrak{s}), \mathbb{A_\beta}(\mathfrak{s}), \mathbb{I}_p(\mathfrak{s}), \mathbb{I}_a(\mathfrak{s}), \mathbb{S}(\mathfrak{s}), \mathbb{D}(\mathfrak{s})) \mathrm{d} \mathfrak{s},  \\ 		\mathbb{I}_{p_{m+1}}=\mathbb{I}_{p_0}+\frac{\mathrm{q}}{\Gamma(\mathrm{p})} \int_0^{\mathrm{t_{m+1}}} \mathfrak{s}^{\mathrm{q}-1}\left(\mathrm{t_{m+1}}-\mathfrak{s}\right)^{\mathrm{p}-1} h_4(\mathfrak{s}, \mathbb{R}(\mathfrak{s}), \mathbb{Q}(\mathfrak{s}), \mathbb{A_\beta}(\mathfrak{s}), \mathbb{I}_p(\mathfrak{s}), \mathbb{I}_a(\mathfrak{s}), \mathbb{S}(\mathfrak{s}), \mathbb{D}(\mathfrak{s})) \mathrm{d} \mathfrak{s}, \\ 		\mathbb{I}_{a_{m+1}}=\mathbb{I}_{a_0}+\frac{\mathrm{q}}{\Gamma(\mathrm{p})} \int_0^{\mathrm{t_{m+1}}} \mathfrak{s}^{\mathrm{q}-1}\left(\mathrm{t_{m+1}}-\mathfrak{s}\right)^{\mathrm{p}-1} h_5(\mathfrak{s}, \mathbb{R}(\mathfrak{s}), \mathbb{Q}(\mathfrak{s}), \mathbb{A_\beta}(\mathfrak{s}), \mathbb{I}_p(\mathfrak{s}), \mathbb{I}_a(\mathfrak{s}), \mathbb{S}(\mathfrak{s}), \mathbb{D}(\mathfrak{s})) \mathrm{d} \mathfrak{s},  \\ 		\mathbb{S}_{m+1}=\mathbb{S}_0+\frac{\mathrm{q}}{\Gamma(\mathrm{p})} \int_0^{\mathrm{t_{m+1}}} \mathfrak{s}^{\mathrm{q}-1}\left(\mathrm{t_{m+1}}-\mathfrak{s}\right)^{\mathrm{p}-1} h_6(\mathfrak{s}, \mathbb{R}(\mathfrak{s}), \mathbb{Q}(\mathfrak{s}), \mathbb{A_\beta}(\mathfrak{s}), \mathbb{I}_p(\mathfrak{s}), \mathbb{I}_a(\mathfrak{s}), \mathbb{S}(\mathfrak{s}), \mathbb{D}(\mathfrak{s})) \mathrm{d} \mathfrak{s},  \\ 		\mathbb{D}_{m+1}=\mathbb{D}_0+\frac{\mathrm{q}}{\Gamma(\mathrm{p})} \int_0^{\mathrm{t_{m+1}}} \mathfrak{s}^{\mathrm{q}-1}\left(\mathrm{t_{m+1}}-\mathfrak{s}\right)^{\mathrm{p}-1} h_7(\mathfrak{s}, \mathbb{R}(\mathfrak{s}), \mathbb{Q}(\mathfrak{s}), \mathbb{A_\beta}(\mathfrak{s}), \mathbb{I}_p(\mathfrak{s}), \mathbb{I}_a(\mathfrak{s}), \mathbb{S}(\mathfrak{s}), \mathbb{D}(\mathfrak{s})) \mathrm{d} \mathfrak{s},  	\end{array}\right. 	$$

Next, the above obtained integrals are approximated as follows:

5.2 \begin{equation*} 		\left\{\begin{array}{l} 			\mathbb{R}_{m+1}=\mathbb{R}_0+\frac{\mathrm{q}}{\Gamma(\mathrm{p})} \sum\limits_{n=0}^{m}\int_{t_n}^{\mathrm{t_{n+1}}} \mathfrak{s}^{\mathrm{q}-1}\left(\mathrm{t_{m+1}}-\mathfrak{s}\right)^{\mathrm{p}-1} h_1(\mathfrak{s}, \mathbb{R}(\mathfrak{s}), \mathbb{Q}(\mathfrak{s}), \mathbb{A_\beta}(\mathfrak{s}), \mathbb{I}_p(\mathfrak{s}), \mathbb{I}_a(\mathfrak{s}), \mathbb{S}(\mathfrak{s}), \mathbb{D}(\mathfrak{s})) \mathrm{d} \mathfrak{s},  \\ 			\mathbb{Q}_{m+1}=\mathbb{Q}_0+\frac{\mathrm{q}}{\Gamma(\mathrm{p})} \sum\limits_{n=0}^{m}\int_{t_n}^{\mathrm{t_{n+1}}} \mathfrak{s}^{\mathrm{q}-1}\left(\mathrm{t_{m+1}}-\mathfrak{s}\right)^{\mathrm{p}-1} h_2(\mathfrak{s}, \mathbb{R}(\mathfrak{s}), \mathbb{Q}(\mathfrak{s}), \mathbb{A_\beta}(\mathfrak{s}), \mathbb{I}_p(\mathfrak{s}), \mathbb{I}_a(\mathfrak{s}), \mathbb{S}(\mathfrak{s}), \mathbb{D}(\mathfrak{s})) \mathrm{d} \mathfrak{s},  \\ 			\mathbb{A_\beta}_{m+1}=\mathbb{A_\beta}_0+\frac{\mathrm{q}}{\Gamma(\mathrm{p})} \sum\limits_{n=0}^{m}\int_{t_n}^{\mathrm{t_{n+1}}} \mathfrak{s}^{\mathrm{q}-1}\left(\mathrm{t_{m+1}}-\mathfrak{s}\right)^{\mathrm{p}-1} h_3(\mathfrak{s}, \mathbb{R}(\mathfrak{s}), \mathbb{Q}(\mathfrak{s}), \mathbb{A_\beta}(\mathfrak{s}), \mathbb{I}_p(\mathfrak{s}), \mathbb{I}_a(\mathfrak{s}), \mathbb{S}(\mathfrak{s}), \mathbb{D}(\mathfrak{s})) \mathrm{d} \mathfrak{s},  \\ 			\mathbb{I}_{p_{m+1}}=\mathbb{I}_{p_0}+\frac{\mathrm{q}}{\Gamma(\mathrm{p})} \sum\limits_{n=0}^{m}\int_{t_n}^{\mathrm{t_{n+1}}} \mathfrak{s}^{\mathrm{q}-1}\left(\mathrm{t_{m+1}}-\mathfrak{s}\right)^{\mathrm{p}-1} h_4(\mathfrak{s}, \mathbb{R}(\mathfrak{s}), \mathbb{Q}(\mathfrak{s}), \mathbb{A_\beta}(\mathfrak{s}), \mathbb{I}_p(\mathfrak{s}), \mathbb{I}_a(\mathfrak{s}), \mathbb{S}(\mathfrak{s}), \mathbb{D}(\mathfrak{s})) \mathrm{d} \mathfrak{s}, \\ 			\mathbb{I}_{a_{m+1}}=\mathbb{I}_{a_0}+\frac{\mathrm{q}}{\Gamma(\mathrm{p})} \sum\limits_{n=0}^{m}\int_{t_n}^{\mathrm{t_{n+1}}} \mathfrak{s}^{\mathrm{q}-1}\left(\mathrm{t_{m+1}}-\mathfrak{s}\right)^{\mathrm{p}-1} h_5(\mathfrak{s}, \mathbb{R}(\mathfrak{s}), \mathbb{Q}(\mathfrak{s}), \mathbb{A_\beta}(\mathfrak{s}), \mathbb{I}_p(\mathfrak{s}), \mathbb{I}_a(\mathfrak{s}), \mathbb{S}(\mathfrak{s}), \mathbb{D}(\mathfrak{s})) \mathrm{d} \mathfrak{s},  \\ 			\mathbb{S}_{m+1}=\mathbb{S}_0+\frac{\mathrm{q}}{\Gamma(\mathrm{p})} \sum\limits_{n=0}^{m}\int_{t_n}^{\mathrm{t_{n+1}}} \mathfrak{s}^{\mathrm{q}-1}\left(\mathrm{t_{m+1}}-\mathfrak{s}\right)^{\mathrm{p}-1} h_6(\mathfrak{s}, \mathbb{R}(\mathfrak{s}), \mathbb{Q}(\mathfrak{s}), \mathbb{A_\beta}(\mathfrak{s}), \mathbb{I}_p(\mathfrak{s}), \mathbb{I}_a(\mathfrak{s}), \mathbb{S}(\mathfrak{s}), \mathbb{D}(\mathfrak{s})) \mathrm{d} \mathfrak{s},  \\ 			\mathbb{D}_{m+1}=\mathbb{D}_0+\frac{\mathrm{q}}{\Gamma(\mathrm{p})} \sum\limits_{n=0}^{m}\int_{t_n}^{\mathrm{t_{n+1}}} \mathfrak{s}^{\mathrm{q}-1}\left(\mathrm{t_{m+1}}-\mathfrak{s}\right)^{\mathrm{p}-1} h_7(\mathfrak{s}, \mathbb{R}(\mathfrak{s}), \mathbb{Q}(\mathfrak{s}), \mathbb{A_\beta}(\mathfrak{s}), \mathbb{I}_p(\mathfrak{s}), \mathbb{I}_a(\mathfrak{s}), \mathbb{S}(\mathfrak{s}), \mathbb{D}(\mathfrak{s})) \mathrm{d} \mathfrak{s},  		\end{array}\right. 	\end{equation*}

By Lagrangian piece-wise interpolation and by applying $l=\mathrm{t}_{\mathrm{n}}-\mathrm{t}_{\mathrm{n}-1}$, within $\left[\mathrm{t}_{\mathrm{m}}, \mathrm{t}_{\mathrm{m}+1}\right]$, we approximate the function $\mathfrak{s}^{\mathrm{q}-1} h_j(\mathfrak{s}, \mathbb{R}, \mathbb{Q}, \mathbb{A_\beta}, \mathbb{I}_p, \mathbb{I}_a, \mathbb{S}, \mathbb{D})$ where $j=1,2, \ldots, 7$, as follows

5.3 \begin{equation*} 		\begin{aligned} 			& \mathbb{R}_{\mathrm{n}}^{\star} \approx \frac{1}{l}\left[\left(\mathrm{t}-\mathrm{t}_{\mathrm{n}-1}\right) \mathrm{t}_{\mathrm{n}}^{\mathrm{q}-1} h_1\left(\mathrm{t}_{\mathrm{n}}, \mathbb{R}_{\mathrm{n}}, \mathbb{Q}_{\mathrm{n}}, \mathbb{A_\beta}_{\mathrm{n}}, \mathbb{I}_{p_\mathrm{n}}, \mathbb{I}_{a_\mathrm{n}}, \mathbb{S}_{\mathrm{n}}, \mathbb{D}_{\mathrm{n}}\right)\right. \\ 			& \left.-\left(\mathrm{t}-\mathrm{t}_{\mathrm{n}}\right) \mathrm{t}_{\mathrm{n}-1}^{\mathrm{q}-1} h_1\left(\mathrm{t}_{\mathrm{n}-1}, \mathbb{R}_{\mathrm{n}-1}, \mathbb{Q}_{\mathrm{n}-1}, \mathbb{A_\beta}_{\mathrm{n}-1}, \mathbb{I}_{p_{\mathrm{n}-1}}, \mathbb{I}_{a_{\mathrm{m}-1}}, \mathbb{S}_{\mathrm{n}-1}, \mathbb{D}_{\mathrm{n}-1}\right)\right], \\ 			& \mathbb{Q}_{\mathrm{n}}^{\star} \approx \frac{1}{l}\left[\left(\mathrm{t}-\mathrm{t}_{\mathrm{n}-1}\right) \mathrm{t}_{\mathrm{n}}^{\mathrm{q}-1} h_2\left(\mathrm{t}_{\mathrm{n}}, \mathbb{R}_{\mathrm{n}}, \mathbb{Q}_{\mathrm{n}}, \mathbb{A_\beta}_{\mathrm{n}}, \mathbb{I}_{p_\mathrm{n}}, \mathbb{I}_{a_\mathrm{n}}, \mathbb{S}_{\mathrm{n}}, \mathbb{D}_{\mathrm{n}}\right)\right. \\ 			& \left.-\left(\mathrm{t}-\mathrm{t}_{\mathrm{n}}\right) \mathrm{t}_{\mathrm{n}-1}^{\mathrm{q}-1} h_2\left(\mathrm{t}_{\mathrm{n}-1}, \mathbb{R}_{\mathrm{n}-1}, \mathbb{Q}_{\mathrm{n}-1}, \mathbb{A_\beta}_{\mathrm{n}-1}, \mathbb{I}_{p_{\mathrm{n}-1}}, \mathbb{I}_{a_{\mathrm{m}-1}}, \mathbb{S}_{\mathrm{n}-1}, \mathbb{D}_{\mathrm{n}-1}\right)\right], \\ 			& \mathbb{A_\beta}_{\mathrm{n}}^{\star} \approx \frac{1}{l}\left[\left(\mathrm{t}-\mathrm{t}_{\mathrm{n}-1}\right) \mathrm{t}_{\mathrm{n}}^{\mathrm{q}-1} h_3\left(\mathrm{t}_{\mathrm{n}}, \mathbb{R}_{\mathrm{n}}, \mathbb{Q}_{\mathrm{n}}, \mathbb{A_\beta}_{\mathrm{n}}, \mathbb{I}_{p_\mathrm{n}}, \mathbb{I}_{a_\mathrm{n}}, \mathbb{S}_{\mathrm{n}}, \mathbb{D}_{\mathrm{n}}\right)\right. \\ 			& \left.-\left(\mathrm{t}-\mathrm{t}_{\mathrm{n}}\right) \mathrm{t}_{\mathrm{n}-1}^{\mathrm{q}-1} h_3\left(\mathrm{t}_{\mathrm{n}-1}, \mathbb{R}_{\mathrm{n}-1}, \mathbb{Q}_{\mathrm{n}-1}, \mathbb{A_\beta}_{\mathrm{n}-1}, \mathbb{I}_{p_{\mathrm{n}-1}}, \mathbb{I}_{a_{\mathrm{m}-1}}, \mathbb{S}_{\mathrm{n}-1}, \mathbb{D}_{\mathrm{n}-1}\right)\right], \\ 			& \mathbb{I}_{p_{\mathrm{n}}}^{\star} \approx \frac{1}{l}\left[\left(\mathrm{t}-\mathrm{t}_{\mathrm{n}-1}\right) \mathrm{t}_{\mathrm{n}}^{\mathrm{q}-1} h_4\left(\mathrm{t}_{\mathrm{n}}, \mathbb{R}_{\mathrm{n}}, \mathbb{Q}_{\mathrm{n}}, \mathbb{A_\beta}_{\mathrm{n}}, \mathbb{I}_{p_\mathrm{n}}, \mathbb{I}_{a_\mathrm{n}}, \mathbb{S}_{\mathrm{n}}, \mathbb{D}_{\mathrm{n}}\right)\right. \\ 			& \left.-\left(\mathrm{t}-\mathrm{t}_{\mathrm{n}}\right) \mathrm{t}_{\mathrm{n}-1}^{\mathrm{q}-1} h_4\left(\mathrm{t}_{\mathrm{n}-1}, \mathbb{R}_{\mathrm{n}-1}, \mathbb{Q}_{\mathrm{n}-1}, \mathbb{A_\beta}_{\mathrm{n}-1}, \mathbb{I}_{p_{\mathrm{n}-1}}, \mathbb{I}_{a_{\mathrm{m}-1}}, \mathbb{S}_{\mathrm{n}-1}, \mathbb{D}_{\mathrm{n}-1}\right)\right], \\ 			& \mathbb{I}_{a_{\mathrm{n}}}^{\star} \approx \frac{1}{l}\left[\left(\mathrm{t}-\mathrm{t}_{\mathrm{n}-1}\right) \mathrm{t}_{\mathrm{n}}^{\mathrm{q}-1} h_5\left(\mathrm{t}_{\mathrm{n}}, \mathbb{R}_{\mathrm{n}}, \mathbb{Q}_{\mathrm{n}}, \mathbb{A_\beta}_{\mathrm{n}}, \mathbb{I}_{p_\mathrm{n}}, \mathbb{I}_{a_\mathrm{n}}, \mathbb{S}_{\mathrm{n}}, \mathbb{D}_{\mathrm{n}}\right)\right. \\ 			& \left.-\left(\mathrm{t}-\mathrm{t}_{\mathrm{n}}\right) \mathrm{t}_{\mathrm{n}-1}^{\mathrm{q}-1} h_5\left(\mathrm{t}_{\mathrm{n}-1}, \mathbb{R}_{\mathrm{n}-1}, \mathbb{Q}_{\mathrm{n}-1}, \mathbb{A_\beta}_{\mathrm{n}-1}, \mathbb{I}_{p_{\mathrm{n}-1}}, \mathbb{I}_{a_{\mathrm{m}-1}}, \mathbb{S}_{\mathrm{n}-1}, \mathbb{D}_{\mathrm{n}-1}\right)\right], \\ 			& \mathbb{S}_{\mathrm{n}}^{\star} \approx \frac{1}{l}\left[\left(\mathrm{t}-\mathrm{t}_{\mathrm{n}-1}\right) \mathrm{t}_{\mathrm{n}}^{\mathrm{q}-1} h_6\left(\mathrm{t}_{\mathrm{n}}, \mathbb{R}_{\mathrm{n}}, \mathbb{Q}_{\mathrm{n}}, \mathbb{A_\beta}_{\mathrm{n}}, \mathbb{I}_{p_\mathrm{n}}, \mathbb{I}_{a_\mathrm{n}}, \mathbb{S}_{\mathrm{n}}, \mathbb{D}_{\mathrm{n}}\right)\right. \\ 			& \left.-\left(\mathrm{t}-\mathrm{t}_{\mathrm{n}}\right) \mathrm{t}_{\mathrm{n}-1}^{\mathrm{q}-1} h_6\left(\mathrm{t}_{\mathrm{n}-1}, \mathbb{R}_{\mathrm{n}-1}, \mathbb{Q}_{\mathrm{n}-1}, \mathbb{A_\beta}_{\mathrm{n}-1}, \mathbb{I}_{p_{\mathrm{n}-1}}, \mathbb{I}_{a_{\mathrm{m}-1}}, \mathbb{S}_{\mathrm{n}-1}, \mathbb{D}_{\mathrm{n}-1}\right)\right], \\ 			& \mathbb{D}_{\mathrm{n}}^{\star} \approx \frac{1}{l}\left[\left(\mathrm{t}-\mathrm{t}_{\mathrm{n}-1}\right) \mathrm{t}_{\mathrm{n}}^{\mathrm{q}-1} h_7\left(\mathrm{t}_{\mathrm{n}}, \mathbb{R}_{\mathrm{n}}, \mathbb{Q}_{\mathrm{n}}, \mathbb{A_\beta}_{\mathrm{n}}, \mathbb{I}_{p_\mathrm{n}}, \mathbb{I}_{a_\mathrm{n}}, \mathbb{S}_{\mathrm{n}}, \mathbb{D}_{\mathrm{n}}\right)\right. \\ 			& \left.-\left(\mathrm{t}-\mathrm{t}_{\mathrm{n}}\right) \mathrm{t}_{\mathrm{n}-1}^{\mathrm{q}-1} h_7\left(\mathrm{t}_{\mathrm{n}-1}, \mathbb{R}_{\mathrm{n}-1}, \mathbb{Q}_{\mathrm{n}-1}, \mathbb{A_\beta}_{\mathrm{n}-1}, \mathbb{I}_{p_{\mathrm{n}-1}}, \mathbb{I}_{a_{\mathrm{m}-1}}, \mathbb{S}_{\mathrm{n}-1}, \mathbb{D}_{\mathrm{n}-1}\right)\right], \\ 			& 		\end{aligned} 	\end{equation*}

Using equation (5.3) into (5.2), we obtain

5.4 \begin{equation*} 		\left\{\begin{array}{l} 			\mathbb{R}_{\mathrm{m}+1}=\mathbb{R}_0+\frac{\mathrm{q}}{\Gamma(\mathrm{p})}\sum\limits_{n=0}^{m} \int_{t_n}^{\mathrm{t}_{\mathrm{n}+1}} \mathfrak{s}^{\mathrm{q}-1}\left(\mathrm{t}_{\mathrm{m}+1}-\mathrm{\mathfrak{s}}\right)^{\mathrm{p}-1}\mathbb{R}_{\mathrm{n}}^{\star}(\mathfrak{s}) \mathrm{d}\mathfrak{s}, \\ 			\mathbb{Q}_{\mathrm{m}+1}=\mathbb{Q}_0+\frac{\mathrm{q}}{\Gamma(\mathrm{p})}\sum\limits_{n=0}^{m} \int_{t_n}^{\mathrm{t}_{\mathrm{n}+1}} \mathfrak{s}^{\mathrm{q}-1}\left(\mathrm{t}_{\mathrm{m}+1}-\mathfrak{s}\right)^{\mathrm{p}-1}\mathbb{Q}_{\mathrm{n}}^{\star}(\mathfrak{s}) \mathrm{d}\mathfrak{s}, \\ 			\mathbb{A_\beta}_{\mathrm{m}+1}=\mathbb{A_\beta}_0+\frac{\mathrm{q}}{\Gamma(\mathrm{p})}\sum\limits_{n=0}^{m} \int_{t_n}^{\mathrm{t}_{\mathrm{n}+1}} \mathfrak{s}^{\mathrm{q}-1}\left(\mathrm{t}_{\mathrm{m}+1}-\mathfrak{s}\right)^{\mathrm{p}-1}\mathbb{A_\beta}_{\mathrm{n}}^{\star}(\mathfrak{s}) \mathrm{d}\mathfrak{s}, \\ 			\mathbb{I}_{p_{\mathrm{m}+1}}=\mathbb{I}_{p_0}+\frac{\mathrm{q}}{\Gamma(\mathrm{p})}\sum\limits_{n=0}^{m} \int_{t_n}^{\mathrm{t}_{\mathrm{n}+1}} \mathfrak{s}^{\mathrm{q}-1}\left(\mathrm{t}_{\mathrm{m}+1}-\mathfrak{s}\right)^{\mathrm{p}-1}\mathbb{I}_{p_{\mathrm{n}}}^{\star}(\mathfrak{s}) \mathrm{d}\mathfrak{s}, \\ 			\mathbb{I}_{a_{\mathrm{m}+1}}=\mathbb{I}_{a_0}+\frac{\mathrm{q}}{\Gamma(\mathrm{p})}\sum\limits_{n=0}^{m} \int_{t_n}^{\mathrm{t}_{\mathrm{n}+1}} \mathfrak{s}^{\mathrm{q}-1}\left(\mathrm{t}_{\mathrm{m}+1}-\mathfrak{s}\right)^{\mathrm{p}-1}\mathbb{I}_{a_{\mathrm{n}}}^{\star}(\mathfrak{s}) \mathrm{d}\mathfrak{s}, \\ 			\mathbb{S}_{\mathrm{m}+1}=\mathbb{S}_0+\frac{\mathrm{q}}{\Gamma(\mathrm{p})}\sum\limits_{n=0}^{m} \int_{t_n}^{\mathrm{t}_{\mathrm{n}+1}} \mathfrak{s}^{\mathrm{q}-1}\left(\mathrm{t}_{\mathrm{m}+1}-\mathfrak{s}\right)^{\mathrm{p}-1}\mathbb{S}_{\mathrm{n}}^{\star}(\mathfrak{s}) \mathrm{d}\mathfrak{s}, \\ 			\mathbb{D}_{\mathrm{m}+1}=\mathbb{D}_0+\frac{\mathrm{q}}{\Gamma(\mathrm{p})}\sum\limits_{n=0}^{m} \int_{t_n}^{\mathrm{t}_{\mathrm{n}+1}} \mathfrak{s}^{\mathrm{q}-1}\left(\mathrm{t}_{\mathrm{m}+1}-\mathfrak{s}\right)^{\mathrm{p}-1}\mathbb{D}_{\mathrm{n}}^{\star}(\mathfrak{s}) \mathrm{d}\mathfrak{s}. 		\end{array}\right. 	\end{equation*}

By simplifying the integrals of (5.4), the numerical method of system (2.1) can be determined by using fractal-fractional derivatives in the Caputo form ,as follows

$$ 	\begin{aligned} 		& \mathbb{R}_{\mathrm{m}+1}=\mathbb{R}_0+\frac{\mathrm{q}l^\mathrm{p}}{\Gamma(\mathrm{p}+2)} \sum\limits_{\mathrm{n}=0}^{\mathrm{m}}\left[\mathrm { t } _ { \mathrm { n } } ^ { \mathrm { q} - 1 } h _ { 1 } ( \mathrm { t } _ { \mathrm { n } } , \mathbb { R } _ { \mathrm { n } } , \mathbb { Q } _ { \mathrm { n } } , \mathbb { A_\beta } _ { \mathrm { n } } , \mathbb { I } _ {p_{ \mathrm { n }} } , \mathbb { I } _ { a_{\mathrm { n }} } , \mathbb { S } _ { \mathrm { n } } , \mathbb { D } _ { \mathrm { n } } ) \left((\mathrm{m}+\mathrm{p}+2-\mathrm{n})(1+\mathrm{m}-\mathrm{n})^{\mathrm{p}}\right.\right. \\ 		& \left.-(\mathrm{m}+2 \mathrm{p}+2-\mathrm{n})(\mathrm{m}-\mathrm{n})^{\mathrm{p}}\right)-\mathrm{t}_{\mathrm{n}-1}^{\mathrm{q}-1} h_1\left(\mathrm{t}_{\mathrm{n}-1}, \mathbb{R}_{\mathrm{n}-1}, \mathbb{Q}_{\mathrm{n}-1}, \mathbb{A_\beta}_{\mathrm{n}-1}, \mathbb{I}_{p_{\mathrm{m}-1}}, \mathbb{I}_{a_{\mathrm{m}-1}}, \mathbb{S}_{\mathrm{n}-1}, \mathbb{D}_{\mathrm{n}-1}\right) \\ 		& \left.\times\left((\mathrm{m}+1-\mathrm{n})^{\mathrm{p}+1}-(\mathrm{m}+1+\mathrm{p}-\mathrm{n})(\mathrm{m}-\mathrm{n})^\mathrm{p}\right)\right] \text {, } \\ 		&  		\mathbb{Q}_{\mathrm{m}+1}=\mathbb{Q}_0+\frac{\mathrm{q}l^\mathrm{p}}{\Gamma(\mathrm{p}+2)} \sum\limits_{\mathrm{n}=0}^{\mathrm{m}}\left[\mathrm { t } _ { \mathrm { n } } ^ { \mathrm { q} - 1 } h _ { 2 } ( \mathrm { t } _ { \mathrm { n } } , \mathbb { R } _ { \mathrm { n } } , \mathbb { Q } _ { \mathrm { n } } , \mathbb { A_\beta } _ { \mathrm { n } } , \mathbb { I } _ {p_{ \mathrm { n }} } , \mathbb { I } _ { a_{\mathrm { n }} } , \mathbb { S } _ { \mathrm { n } } , \mathbb { D } _ { \mathrm { n } } ) \left((\mathrm{m}+\mathrm{p}+2-\mathrm{n})(1+\mathrm{m}-\mathrm{n})^{\mathrm{p}}\right.\right. \\ 		& \left.-(\mathrm{m}+2 \mathrm{p}+2-\mathrm{n})(\mathrm{m}-\mathrm{n})^{\mathrm{p}}\right)-\mathrm{t}_{\mathrm{n}-1}^{\mathrm{q}-1} h_2\left(\mathrm{t}_{\mathrm{n}-1}, \mathbb{R}_{\mathrm{n}-1}, \mathbb{Q}_{\mathrm{n}-1}, \mathbb{A_\beta}_{\mathrm{n}-1}, \mathbb{I}_{p_{\mathrm{m}-1}}, \mathbb{I}_{a_{\mathrm{m}-1}}, \mathbb{S}_{\mathrm{n}-1}, \mathbb{D}_{\mathrm{n}-1}\right) \\ 		& \left.\times\left((\mathrm{m}+1-\mathrm{n})^{\mathrm{p}+1}-(\mathrm{m}+1+\mathrm{p}-\mathrm{n})(\mathrm{m}-\mathrm{n})^\mathrm{p}\right)\right] \text {, } \\ 		& 		\mathbb{A_\beta}_{\mathrm{m}+1}=\mathbb{A_\beta}_0+\frac{\mathrm{q}l^\mathrm{p}}{\Gamma(\mathrm{p}+2)} \sum\limits_{\mathrm{n}=0}^{\mathrm{m}}\left[\mathrm { t } _ { \mathrm { n } } ^ { \mathrm { q} - 1 } h _ { 3 } ( \mathrm { t } _ { \mathrm { n } } , \mathbb { R } _ { \mathrm { n } } , \mathbb { Q } _ { \mathrm { n } } , \mathbb { A_\beta } _ { \mathrm { n } } , \mathbb { I } _ {p_{ \mathrm { n }} } , \mathbb { I } _ { a_{\mathrm { n }} } , \mathbb { S } _ { \mathrm { n } } , \mathbb { D } _ { \mathrm { n } } ) \left((\mathrm{m}+\mathrm{p}+2-\mathrm{n})(1+\mathrm{m}-\mathrm{n})^{\mathrm{p}}\right.\right. \\ 		& \left.-(\mathrm{m}+2 \mathrm{p}+2-\mathrm{n})(\mathrm{m}-\mathrm{n})^{\mathrm{p}}\right)-\mathrm{t}_{\mathrm{n}-1}^{\mathrm{q}-1} h_3\left(\mathrm{t}_{\mathrm{n}-1}, \mathbb{R}_{\mathrm{n}-1}, \mathbb{Q}_{\mathrm{n}-1}, \mathbb{A_\beta}_{\mathrm{n}-1}, \mathbb{I}_{p_{\mathrm{m}-1}}, \mathbb{I}_{a_{\mathrm{m}-1}}, \mathbb{S}_{\mathrm{n}-1}, \mathbb{D}_{\mathrm{n}-1}\right) \\ 		& \left.\times\left((\mathrm{m}+1-\mathrm{n})^{\mathrm{p}+1}-(\mathrm{m}+1+\mathrm{p}-\mathrm{n})(\mathrm{m}-\mathrm{n})^\mathrm{p}\right)\right] \text {, } \\ 		& 		\mathbb{I}_{p_{\mathrm{m}+1}}=\mathbb{I}_{p_0}+\frac{\mathrm{q}l^\mathrm{p}}{\Gamma(\mathrm{p}+2)} \sum\limits_{\mathrm{n}=0}^{\mathrm{m}}\left[\mathrm { t } _ { \mathrm { n } } ^ { \mathrm { q} - 1 } h _ { 4 } ( \mathrm { t } _ { \mathrm { n } } , \mathbb { R } _ { \mathrm { n } } , \mathbb { Q } _ { \mathrm { n } } , \mathbb { A_\beta } _ { \mathrm { n } } , \mathbb { I } _ {p_{ \mathrm { n }} } , \mathbb { I } _ { a_{\mathrm { n }} } , \mathbb { S } _ { \mathrm { n } } , \mathbb { D } _ { \mathrm { n } } ) \left((\mathrm{m}+\mathrm{p}+2-\mathrm{n})(1+\mathrm{m}-\mathrm{n})^{\mathrm{p}}\right.\right. \\ 		& \left.-(\mathrm{m}+2 \mathrm{p}+2-\mathrm{n})(\mathrm{m}-\mathrm{n})^{\mathrm{p}}\right)-\mathrm{t}_{\mathrm{n}-1}^{\mathrm{q}-1} h_4\left(\mathrm{t}_{\mathrm{n}-1}, \mathbb{R}_{\mathrm{n}-1}, \mathbb{Q}_{\mathrm{n}-1}, \mathbb{A_\beta}_{\mathrm{n}-1}, \mathbb{I}_{p_{\mathrm{m}-1}}, \mathbb{I}_{a_{\mathrm{m}-1}}, \mathbb{S}_{\mathrm{n}-1}, \mathbb{D}_{\mathrm{n}-1}\right) \\ 		&\left.\times\left((\mathrm{m}+1-\mathrm{n})^{\mathrm{p}+1}-(\mathrm{m}+1+\mathrm{p}-\mathrm{n})(\mathrm{m}-\mathrm{n})^\mathrm{p}\right)\right] \text {, } \\ 		& 		\mathbb{I}_{a_{\mathrm{m}+1}}=\mathbb{I}_{a_0}+\frac{\mathrm{q}l^\mathrm{p}}{\Gamma(\mathrm{p}+2)} \sum\limits_{\mathrm{n}=0}^{\mathrm{m}}\left[\mathrm { t } _ { \mathrm { n } } ^ { \mathrm { q} - 1 } h _ { 5 } ( \mathrm { t } _ { \mathrm { n } } , \mathbb { R } _ { \mathrm { n } } , \mathbb { Q } _ { \mathrm { n } } , \mathbb { A_\beta } _ { \mathrm { n } } , \mathbb { I } _ {p_{ \mathrm { n }} } , \mathbb { I } _ { a_{\mathrm { n }} } , \mathbb { S } _ { \mathrm { n } } , \mathbb { D } _ { \mathrm { n } } ) \left((\mathrm{m}+\mathrm{p}+2-\mathrm{n})(1+\mathrm{m}-\mathrm{n})^{\mathrm{p}}\right.\right. \\ 		& \left.-(\mathrm{m}+2 \mathrm{p}+2-\mathrm{n})(\mathrm{m}-\mathrm{n})^{\mathrm{p}}\right)-\mathrm{t}_{\mathrm{n}-1}^{\mathrm{q}-1} h_5\left(\mathrm{t}_{\mathrm{n}-1}, \mathbb{R}_{\mathrm{n}-1}, \mathbb{Q}_{\mathrm{n}-1}, \mathbb{A_\beta}_{\mathrm{n}-1}, \mathbb{I}_{p_{\mathrm{m}-1}}, \mathbb{I}_{a_{\mathrm{m}-1}}, \mathbb{S}_{\mathrm{n}-1}, \mathbb{D}_{\mathrm{n}-1}\right) \\ 		& \left.\times\left((\mathrm{m}+1-\mathrm{n})^{\mathrm{p}+1}-(\mathrm{m}+1+\mathrm{p}-\mathrm{n})(\mathrm{m}-\mathrm{n})^\mathrm{p}\right)\right] \text {, } \\ 		& 		\mathbb{S}_{\mathrm{m}+1}=\mathbb{S}_0+\frac{\mathrm{q}l^\mathrm{p}}{\Gamma(\mathrm{p}+2)} \sum\limits_{\mathrm{n}=0}^{\mathrm{m}}\left[\mathrm { t } _ { \mathrm { n } } ^ { \mathrm { q} - 1 } h _ { 6 } ( \mathrm { t } _ { \mathrm { n } } , \mathbb { R } _ { \mathrm { n } } , \mathbb { Q } _ { \mathrm { n } } , \mathbb { A_\beta } _ { \mathrm { n } } , \mathbb { I } _ {p_{ \mathrm { n }} } , \mathbb { I } _ { a_{\mathrm { n }} } , \mathbb { S } _ { \mathrm { n } } , \mathbb { D } _ { \mathrm { n } } ) \left((\mathrm{m}+\mathrm{p}+2-\mathrm{n})(1+\mathrm{m}-\mathrm{n})^{\mathrm{p}}\right.\right. \\ 		& \left.-(\mathrm{m}+2 \mathrm{p}+2-\mathrm{n})(\mathrm{m}-\mathrm{n})^{\mathrm{p}}\right)-\mathrm{t}_{\mathrm{n}-1}^{\mathrm{q}-1} h_6\left(\mathrm{t}_{\mathrm{n}-1}, \mathbb{R}_{\mathrm{n}-1}, \mathbb{Q}_{\mathrm{n}-1}, \mathbb{A_\beta}_{\mathrm{n}-1}, \mathbb{I}_{p_{\mathrm{m}-1}}, \mathbb{I}_{a_{\mathrm{m}-1}}, \mathbb{S}_{\mathrm{n}-1}, \mathbb{D}_{\mathrm{n}-1}\right) \\ 		& \left.\times\left((\mathrm{m}+1-\mathrm{n})^{\mathrm{p}+1}-(\mathrm{m}+1+\mathrm{p}-\mathrm{n})(\mathrm{m}-\mathrm{n})^\mathrm{p}\right)\right] \text {, } \\ 		& \mathbb{D}_{\mathrm{m}+1}=\mathbb{D}_0+\frac{\mathrm{q}l^\mathrm{p}}{\Gamma(\mathrm{p}+2)} \sum\limits_{\mathrm{n}=0}^{\mathrm{m}}\left[\mathrm { t } _ { \mathrm { n } } ^ { \mathrm { q} - 1 } h _ { 7 } ( \mathrm { t } _ { \mathrm { n } } , \mathbb { R } _ { \mathrm { n } } , \mathbb { Q } _ { \mathrm { n } } , \mathbb { A_\beta } _ { \mathrm { n } } , \mathbb { I } _ {p_{ \mathrm { n }} } , \mathbb { I } _ { a_{\mathrm { n }} } , \mathbb { S } _ { \mathrm { n } } , \mathbb { D } _ { \mathrm { n } } ) \left((\mathrm{m}+\mathrm{p}+2-\mathrm{n})(1+\mathrm{m}-\mathrm{n})^{\mathrm{p}}\right.\right. \\ 		& \left.-(\mathrm{m}+2 \mathrm{p}+2-\mathrm{n})(\mathrm{m}-\mathrm{n})^{\mathrm{p}}\right)-\mathrm{t}_{\mathrm{n}-1}^{\mathrm{q}-1} h_7\left(\mathrm{t}_{\mathrm{n}-1}, \mathbb{R}_{\mathrm{n}-1}, \mathbb{Q}_{\mathrm{n}-1}, \mathbb{A_\beta}_{\mathrm{n}-1}, \mathbb{I}_{p_{\mathrm{m}-1}}, \mathbb{I}_{a_{\mathrm{m}-1}}, \mathbb{S}_{\mathrm{n}-1}, \mathbb{D}_{\mathrm{n}-1}\right) \\ 		& \left.\times\left((\mathrm{m}+1-\mathrm{n})^{\mathrm{p}+1}-(\mathrm{m}+1+\mathrm{p}-\mathrm{n})(\mathrm{m}-\mathrm{n})^\mathrm{p}\right)\right] \text {, } \\ 		& 	\end{aligned} 	$$

## 6. Numerical simulation and discussion

**Figure 2.**
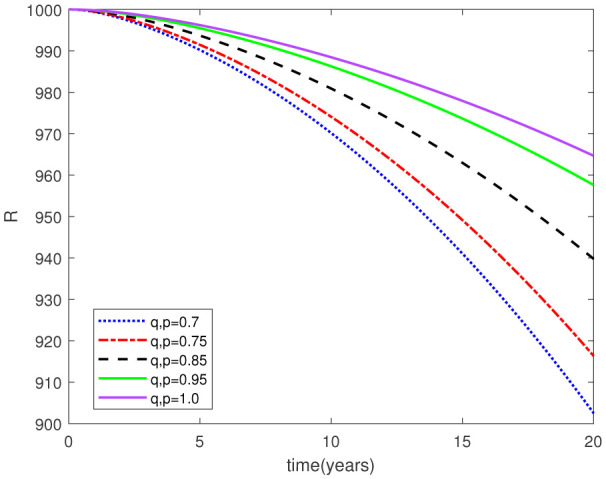
Dynamical variation of proliferative reactive astrocytes of model (2.1) when F-D q and F-O p are equal (p, q ∈ [0, 1]).

**Figure 3.**
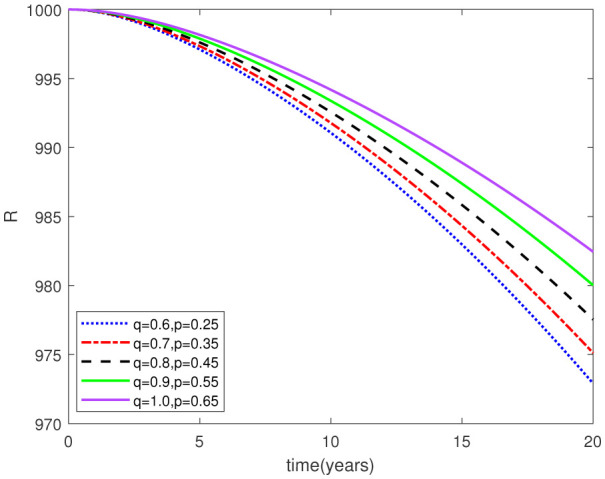
Dynamical variation of proliferative reactive astrocytes of model (2.1) for different F-D q and F-O p (p, q ∈ [0, 1]).

**Figure 4.**
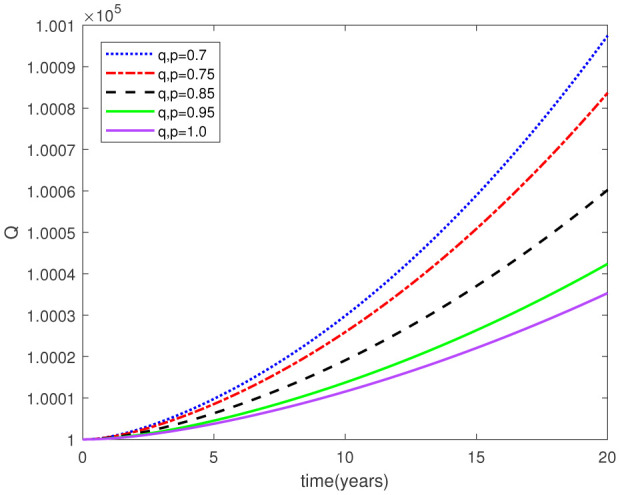
Dynamical variation of quiescent (resting) astrocytes of model (2.1) when F-D q and F-O p are equal (p, q ∈ [0, 1]).

**Figure 5.**
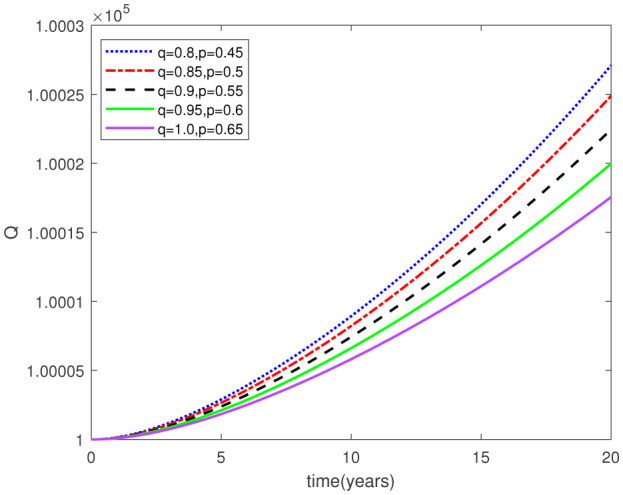
Dynamical variation of quiescent (resting) astrocytes of model (2.1) for different F-D q and F-O p (p, q ∈ [0, 1]).

**Figure 6.**
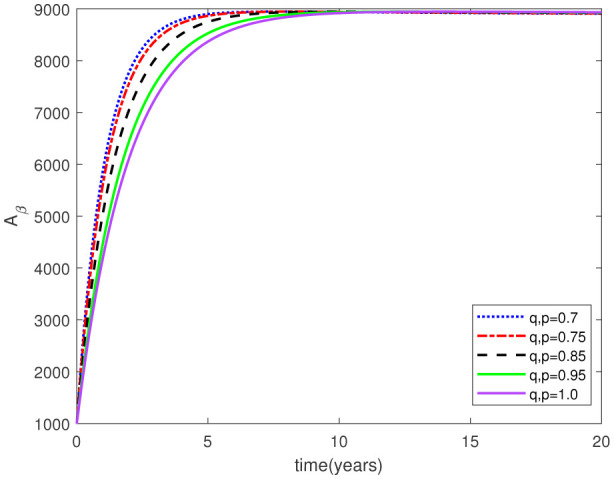
Dynamical variation of aggregation-prone amyloid-*β* fibrils of model (2.1) when F-D q and F-O p are equal (p, q q ∈ [0, 1]).

**Figure 7.**
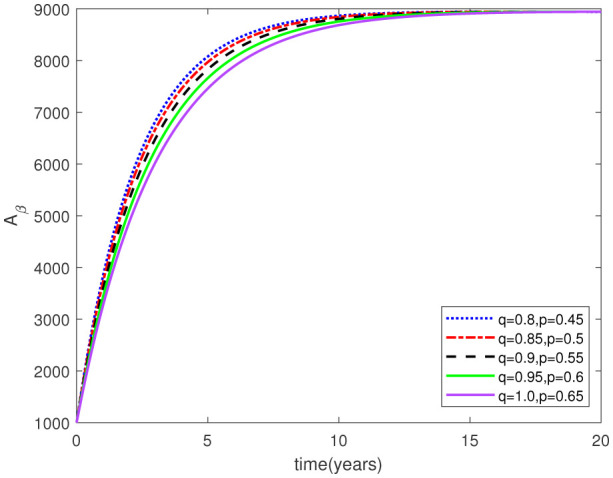
Dynamical variation of aggregation-prone amyloid-*β* fibrils of model (2.1) for different F-D q and F-O p (p, q ∈ [0, 1]).

**Figure 8.**
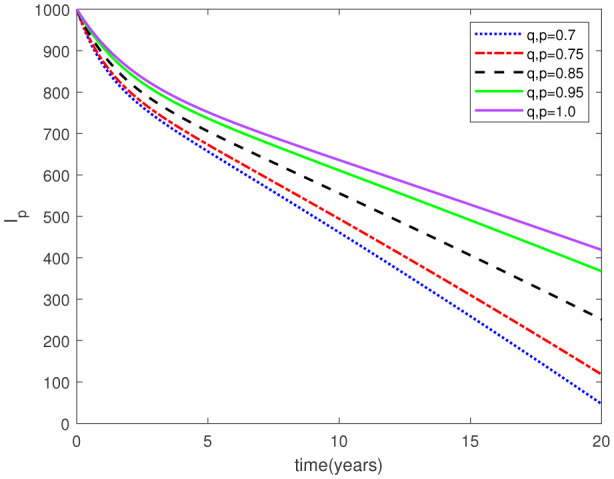
Dynamical variation of activated microglia in anti-inflammatory state of model (2.1) when F-D q and F-O p are equal (p, q ∈ [0, 1]).

**Figure 9.**
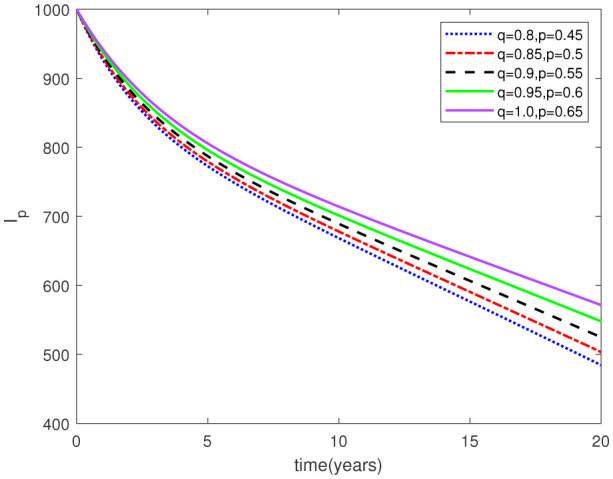
Dynamical variation of activated microglia in anti-inflammatory state of model (2.1) for different F-D q and F-O p (p, q ∈ [0, 1]).

**Figure 10.**
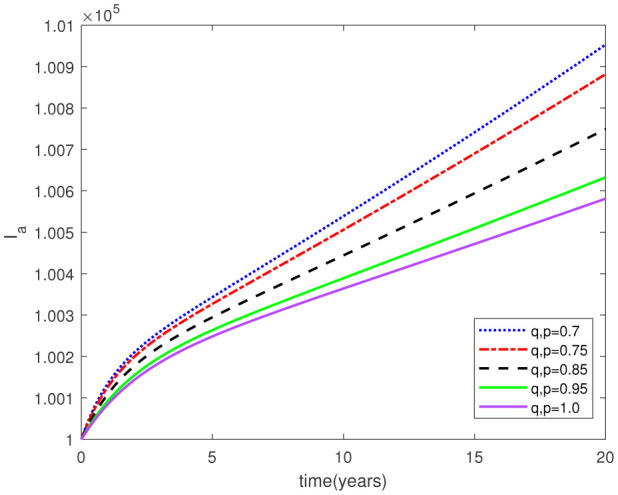
Dynamical variation of activated microglia in pro-inflammatory state of model (2.1) when F-D q and F-O p are equal (p, q ∈ [0, 1]).

**Figure 11.**
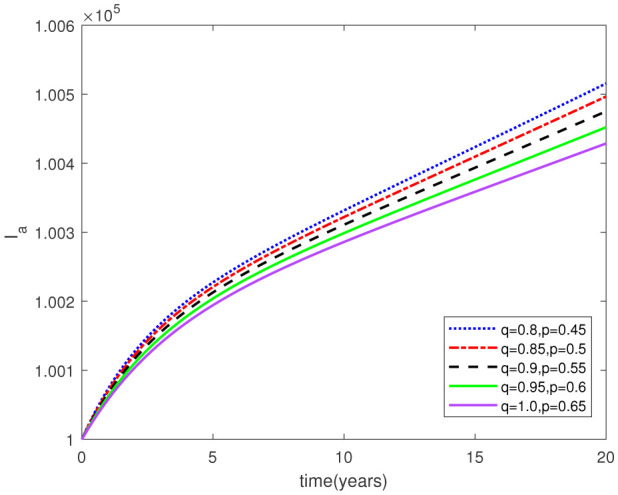
Dynamical variation of activated microglia in pro-inflammatory state of model (2.1) for different F-D q and F-O p (p, q ∈ [0, 1]).

**Figure 12.**
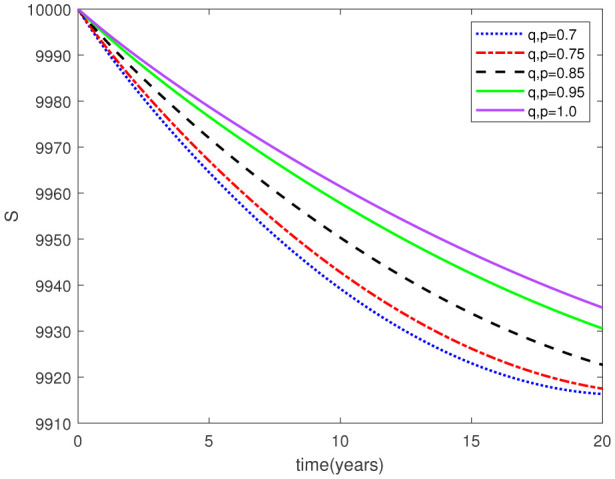
Dynamical variation of surviving neurons of model (2.1) when F-D q and F-O p are equal (p, q ∈ [0, 1]).

**Figure 13.**
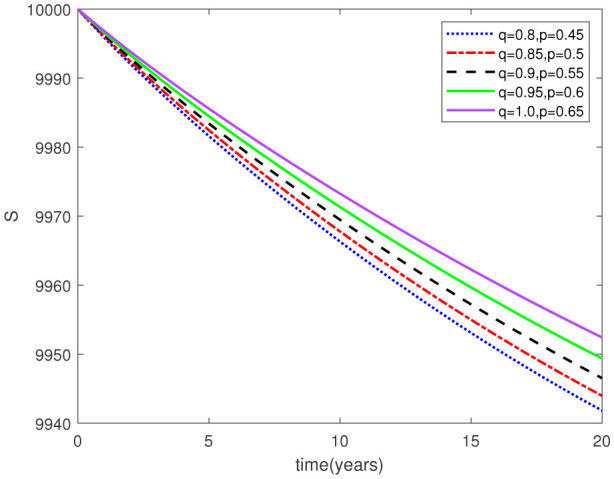
Dynamical variation of surviving neurons of model (2.1) for different F-D q and F-O p (p, q ∈ [0, 1]).

**Figure 14.**
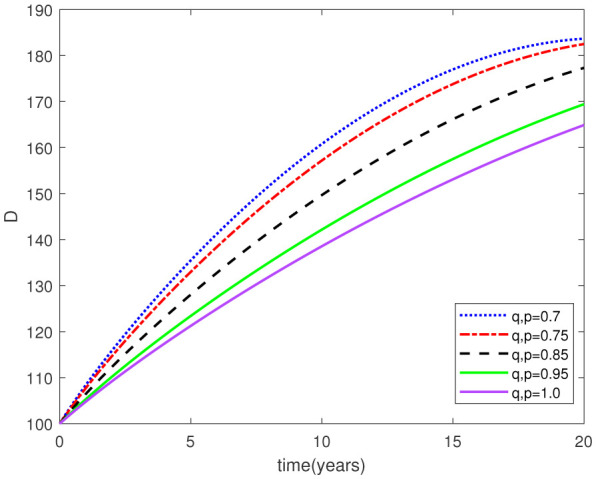
Dynamical variation of dead neurons of model (2.1) when F-D q and F-O p are equal (p, q ∈ [0, 1]).

**Figure 15.**
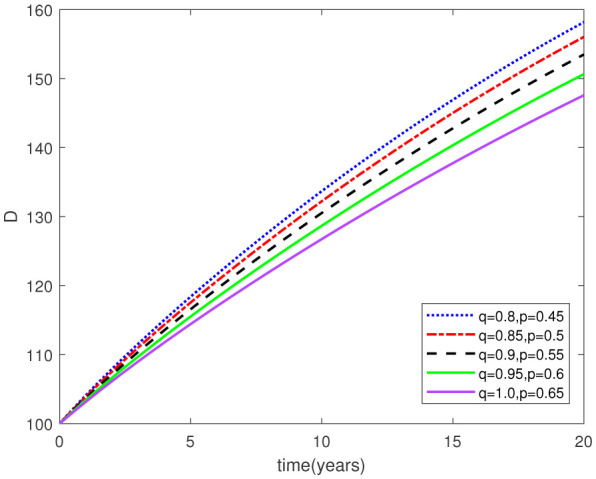
Dynamical variation of dead neurons of model (2.1) for different F-D q and F-O p (p, q ∈ [0, 1]).

This study examined a fractal-fractional type model by employing the Caputo derivative framework to obtain graphical and numerical outcomes. Our approach involved making assumptions about certain parameter values based on the data provided in Table 1 for the model under investigation. Subsequently, we conducted simulations for the relevant compartments of the system (2.1), as depicted in Figures 2-15. These simulations maintained consistent F-O p and F-D q values, while also varying the fractal-fractional orders. The period of time applied for this simulation was 20 years. The following intial conditions were considered from [23]:

$\mathbb{R}(0)=10^3$; $\mathbb{Q}(0)=10^5$; $\mathbb{A}_\beta(0)=10^3$; $\mathbb{I}_p(0)=10^3$; $\mathbb{I}_a(0)=10^5$; $\mathbb{S}(0)=10^4$; $\mathbb{D}(0)=10^2$.

Parameters play a crucial role in the investigation of diseases when employing numerical solutions, and the F-O p and F-D q constitute a key indicator of the optimal memory effect. The population of $\mathbb{ A_\beta }$ fibrils decreases as the F-D q and F-O p values approaches to unity, as depicted in Figures 6 and 7. This reduction correlates with the smallest decrease in the population of surviving neurons $\mathbb{S}$, as shown in Figures 12 and 13. Additionally, there are only slight increments in the populations of $\mathbb{I}_a$ and $\mathbb{Q}$ cells, as shown in Figures 10 and 11 and Figures 4 and 5, respectively. The observation that elevated populations of $\mathbb{ I }_a$ and $\mathbb{Q}$ cells are unable to reduce $\mathbb{ A_\beta }$ fibril levels or cease the progression, and may even aggravate the decline in neuronal populations, suggests a potential association between the interplay of brain cells and $\mathbb{ A_\beta }$ fibrils that leads to anomalous behavior in response to pro-inflammatory mutations associated with age and AD-related chronic inflammation [4], [37]. In accordance with the findings presented in [23], the dynamics of the $\mathbb{ A_\beta }$ fibril population have been mathematically characterized, demonstrating a tendency to reach a saturation point approximately five years after initiation (refer to Figure 6). This phenomenon exerts a moderating influence on the subsequent decline (or increase) in the populations of deceased and survival neurons, during later time intervals (as depicted in Figures 12 and 14). It is worth noting that the temporal fluctuations in the populations of survival neurons bear a resemblance to the observed trends in the Mini-Mental State examination (MMSE) outcomes. These MMSE assessments were administered to individuals afflicted with late-stage mild cognitive impairment, a cerebral degenerative condition that, in certain instances, precedes the onset of AD. This comparative analysis spans a period of ten years (as illustrated in Figure 3c in [36]). Until a definitive remedy for AD becomes available, the integration of anti-inflammatory treatments with a wholesome lifestyle could offer an effective means of enhancing neuroprotection, potentially staving off the initiation of AD or slowing down its advancement. Figures 2, 4, 6, 8, 10, 12 and 14 shows the dynamical variations of the seven populations of the model (2.1) for the same F-D q and the F-O p values for q, p ∈ [0, 1]. Similarly, Figures 3, 5, 7, 9, 11, reffig13 and 15 shows the dynamical variations of the seven populations of the model (2.1) for the different F-D q and the F-O p values for q, p p ∈ [0, 1]. The F-D q and the F-O p assumes a critical role in the simulation experiments for the AD model conducted in this study. In contrast, our study incorporates the fractal fractional order derivative in the Caputo sense to capture the behavior of the AD model. The simulation outcomes demonstrate that even slight adjustments in the F-D q and the F-O p can significantly influence the numerical results. Therefore, when working with experimental studies, it becomes crucial to accurately determine the precise values of the F-D q and the F-O p to attain enhanced precision in the outcomes.

## 7. Conclusion

In this article, the dynamics of AD have been derived by using an arbitrary order differential equation system. A mathematical model of AD has been presented that involves $\mathbb{ A_\beta }$, normal and reactive glial cells and neurons. A new fractal-fractional Caputo derivative approach has been developed and applied to the model (2.1) with F-D q and F-O p. In our research, we have achieved significant progress in advancing the theoretical foundations of the proposed model. Using innovative techniques inspired by the Banach and Schaefer's fixed-point theorems, we have rigorously demonstrated the existence of a unique solution for the model. Furthermore, we have employed nonlinear functional analysis to establish the requisite conditions for Ulam-Hyers stability, thereby confirming the stability of the obtained solution. To validate our findings, we conducted simulations with varying values of p and q by using the fractional type Adams-Bashforth method. All the computational simulations were performed in MATLAB (R2023) and are shown graphically. The model employs fractal fractional derivatives as a means to characterize the temporal dynamics of specific cell populations and the formation of amyloid-*β* fibrils that are prone to aggregation. In the numerical discussion section, we provide two simulation cases to discuss. The first case involves the same F-O and F-D scheme, which we have compared to integer order. The second case features different F-D and F-O, which we also compared to integer-order. This paper opens up avenues for future research and one can explore alternative types of fractal-fractional operators by using real-world data. Further, introducing nonlinearity into the considered system could impact the dynamics of the complex system. We anticipate that this will lead to a more thorough understanding of AD and may reveal fresh insights into its progression and treatment options.

## Use of AI tools declaration

The authors declare they have not used Artificial Intelligence (AI) tools in the creation of this article.
